# An equity-oriented rethink of global rankings with complex networks mapping development

**DOI:** 10.1038/s41598-020-74964-3

**Published:** 2020-10-22

**Authors:** Loredana Bellantuono, Alfonso Monaco, Sabina Tangaro, Nicola Amoroso, Vincenzo Aquaro, Roberto Bellotti

**Affiliations:** 1 Dipartimento Interateneo di Fisica “M. Merlin”, Università degli Studi di Bari “A. Moro”, 70126 Bari, Italy; 2 Istituto Nazionale di Fisica Nucleare, Sezione di Bari, 70125 Bari, Italy; 3 Dipartimento di Scienze del Suolo, della Pianta e degli Alimenti, Università degli Studi di Bari “A. Moro”, 70126 Bari, Italy; 4 Dipartimento di Farmacia-Scienze del Farmaco, Università degli Studi di Bari “A. Moro”, 70125 Bari, Italy; 5 Division for Public Institutions and Digital Government, United Nations Department of Economic and Social Affairs (DESA), New York, NY 10017 USA

**Keywords:** Applied mathematics, Scientific data, Applied physics

## Abstract

Nowadays, world rankings are promoted and used by international agencies, governments and corporations to evaluate country performances in a specific domain, often providing a guideline for decision makers. Although rankings allow a direct and quantitative comparison of countries, sometimes they provide a rather oversimplified representation, in which relevant aspects related to socio-economic development are either not properly considered or still analyzed in silos. In an increasingly data-driven society, a new generation of cutting-edge technologies is breaking data silos, enabling new use of public indicators to generate value for multiple stakeholders. We propose a complex network framework based on publicly available indicators to extract important insight underlying global rankings, thus adding value and significance to knowledge provided by these rankings. This approach enables the unsupervised identification of communities of countries, establishing a more targeted, fair and meaningful criterion to detect similarities. Hence, the performance of states in global rankings can be assessed based on their development level. We believe that these evaluations can be crucial in the interpretation of global rankings, making comparison between countries more significant and useful for citizens and governments and creating ecosystems for new opportunities for development.

## Introduction

In recent years, the Earth and our society have been hit by severe crises on a global scale, in crucial sectors, ranging from health (pandemics) to environment (climate change) and economics (the 2008 crisis)^1–7^. These events have seriously questioned development models, highlighting the need to improve governance systems. In particular, necessity to implement advanced forms of decision-making, oriented at undertaking joint and strategic action by different countries, has emerged^8^. To ensure an effective realization of these projects, a deep knowledge of resources and criticalities of each country, as well as of similarities and differences between countries, is essential. Supranational agencies that assess the status of world countries by exploring in detail specific aspects of development, including health, environment, culture, human rights, innovation, exist since the mid-ninetieth century^9^, the oldest one still active to date being the International Telecommunication Union (ITU, 1865). Since the final stages of WWII, the need for an increased international cooperation triggered the establishment of the United Nations (UN, 1945), which incorporated existing agencies and founded new ones such as the World Health Organization (WHO, 1948) and the United Nations Children’s Fund (UNICEF, 1946), and of independent agencies like the Organisation for Economic Co-operation and Development (OECD, 1948) and the World Bank (1944). Through the years, the aforementioned assessment processes have become more and more widespread, immediate and rigorous, thanks to the increasing availability and accuracy of data, provided either by the same international organizations or by the member countries, and to the application of statistical methods^10^. In recent times, the profitable synergy between political decision-makers and data scientists has allowed to identify a set of priority intervention areas, synthesized in the 2030 Agenda of Sustainable Development Goals (SDGs) in the form of well-defined objectives, whose attainment can be tracked and monitored in a transparent way, through a set of measurable indicators^8^. All countries, regardless of their development level, are called to provide an effort to direct their national development policies towards sustainability. The crucial aspect characterizing SDGs is their universality: each SDG captures problems that involve all countries, highlighting their interdependence, since, in a globalized world, the action of a single country is followed by a spillover effect on the rest. Certainly, the issues targeted by each objective are declined in different ways, according to the current development condition. Therefore, each UN country is committed to define a sustainable development strategy, translating the 2030 Agenda into national embedded plans and policies that are at the same time feasible and ambitious. It is thus crucial to develop rigorous, transparent and replicable methodologies to support states in this process, providing tools for data measurement and benchmarking, prioritization of targets and monitoring of progress, tailored to their specific conditions and needs.

The development degree of a country can be viewed as the result of a plurality of social, economic, cultural, environmental and geopolitical (local and regional) factors, combined with different modalities. If, on one hand, it is appropriate to characterize in a clear and synthetic form the status of a country through aggregate development indexes, on the other hand it is necessary to keep track of the complexity behind. Applying innovative analysis methods, it is possible to combine these two needs. Novel instruments of complexity science have become complementary to traditional statistical tools in assessing the status of a country or territory, integrating conventional approaches and unveiling hidden information. This vision is based on the concept of economic complexity^11^, according to which the ensemble of goods and services produced by a country is the expression of a system of intangible features like capacities, knowledge and skills, that are not captured by standard economic indicators. In this framework, products and services provide the basis for the conceptualization of new metrics (e.g., the economic fitness^12^), able to quantify the competitiveness of both states^13^ and innovation sectors^14^. Analogously, each state owns a heritage of cultural, economic, social and infrastructural peculiarities, that act as development-delivery mechanisms^15^, fostering the transition from rural economies towards industrialization^16^. Non-governmental organizations, public and private research institutes, statistical agencies and foundations have released on public platforms huge quantities of open data, containing indicators on economics, society, development and rights of world countries^17–20^ and of subnational entities^21,22^. These data, being compiled from officially recognized international institutions, provide a huge amount of reliable information, which may aid states and organizations to design plans for resource allocations, and prompted the rise of new trends in policy-making such as new governance^23–25^ and experimentalist governance^26,27^.

A common way of rating the performance of countries with respect to a specific indicator or a group of indicators is to draw up a ranking^13,28–31^, with higher- and lower-performing states at the extremal positions. Indicators and rankings provide a simplified representation of complex cultural, social and economic phenomena, constituting one of the few quantitative means to navigate the uncertainty of complex social systems^32^. The growing use of indicators and rankings for evaluative purpose and in global governance therefore requires the utmost attention in their construction and interpretation, to avoid critical issues. On one hand, composite indexes entail a loss of information and are affected by arbitrariness, biases and possible inaccuracies in the choice and aggregation of different, perhaps inter-related, indicators^33–35^. On the other hand, the interpretation of rankings can be insidious, as they often represent a mere snapshot of the *status-quo* of the considered countries, and do not take into account the development degree behind a given position and the heterogeneity of possible starting conditions. A particularly striking consideration is that rankings tend to emphasize country differences, while similarity should be the driving aspect in country performance assessment^36^. Adding highly detailed information on the development variables helps analysts and decision-makers understanding whether the position of a country in a ranking should be considered positive or not, based on the comparison with other countries, recognized as similar in wide-ranging areas of global development. This approach has advantages in both short- and long-term plans. On one hand, it provides an equity-oriented criterion to evaluate country performances in a given ranking. On the other hand, it reinterprets in an effective way the concept of *proximity* of economic complexity^12,37–40^, according to which it is fundamental to capture similarities between states, to identify and try to enhance possible unexpressed potentialities.

To date, the approach to country performance evaluation has been based on the definition of proxies, namely single or aggregate indicators, correlated with the index of the ranking to be contextualized. A commonly used proxy is GNI per capita^15,41^, but in the last years Socio-Demographic Index (SDI), an aggregate measure which combines indicators related to income, education and total fertility rate^42,43^, has become increasingly employed. In this framework, the performance of a country is assessed upon comparison with an expected value, determined by a regression procedure based on the proxy.

The goal of this work is to establish a robust, insightful and reproducible pipeline to evaluate the performance of a country in rankings, taking into account both its development status and its similarity with other countries. To the best of our knowledge, the proposed strategy represents the first equity-oriented rethink of global rankings based on a complex and multifaceted representation of development, rather than on individual proxies stemming from arbitrarily aggregated indexes. The idea is to adopt the machinery of complex network theory^44^, related, in particular, to community detection^45,46^, to partition world countries in groups according to their development similarities. The complex network formalism is a multidisciplinary tool which has been increasingly used to represent real-world complex systems, consisting of non trivially interconnected constituents. Fertile application fields include economics^14,39,47^, human mobility^48,49^, neuroscience^50–52^, genetics^53^, just to mention a few; moreover, recent studies are endowing the complex network toolbox with new instruments, such as multilayer networks^54^ and network potentials^55^. In particular, the paradigm of multilayer networks has recently been employed to quantify country relevance in international relations^56^, using node centralities as proxies^57^. In our study we shall focus on the identification of network communities, sets of nodes with a tendency to connect among themselves more than with nodes in the complementary set. Community detection provides a way to group countries according to their similarity with respect to a wide range of parameters related to development. This method of classifying countries opens the way to new equity-based evaluation criteria. Actually, the proposed approach provides a key to reinterpret rankings and to identify cases of either leading countries, that reach higher positions compared to the others in the same development class, as well as trailing countries. The evaluations can provide a quantitative basis to diagnose problematic scenarios and enact support policies. The reliability of our evaluation is strengthened by a quantitative control to check that the ranked index distributions related to different network communities are separated in a relevant way, an essential condition to validate the proposed methodology. The UN and the World Bank currently make use, for statistical purposes, of subdivisions of world countries in development groups^58^ and income groups^59^, respectively. These two are prominent examples of world partitions that either focus on a very specific aspect of development or rest on weak analytical foundations^60^. Our model, instead, partitions the world UN states network in an unsupervised way, encompassing, in principle, all possible dimensions of development: community detection keeps track of relevant similarities between countries, which can be sometimes hidden, unexpected and not obtainable from merely geographical and economic considerations.

**Figure 1:**
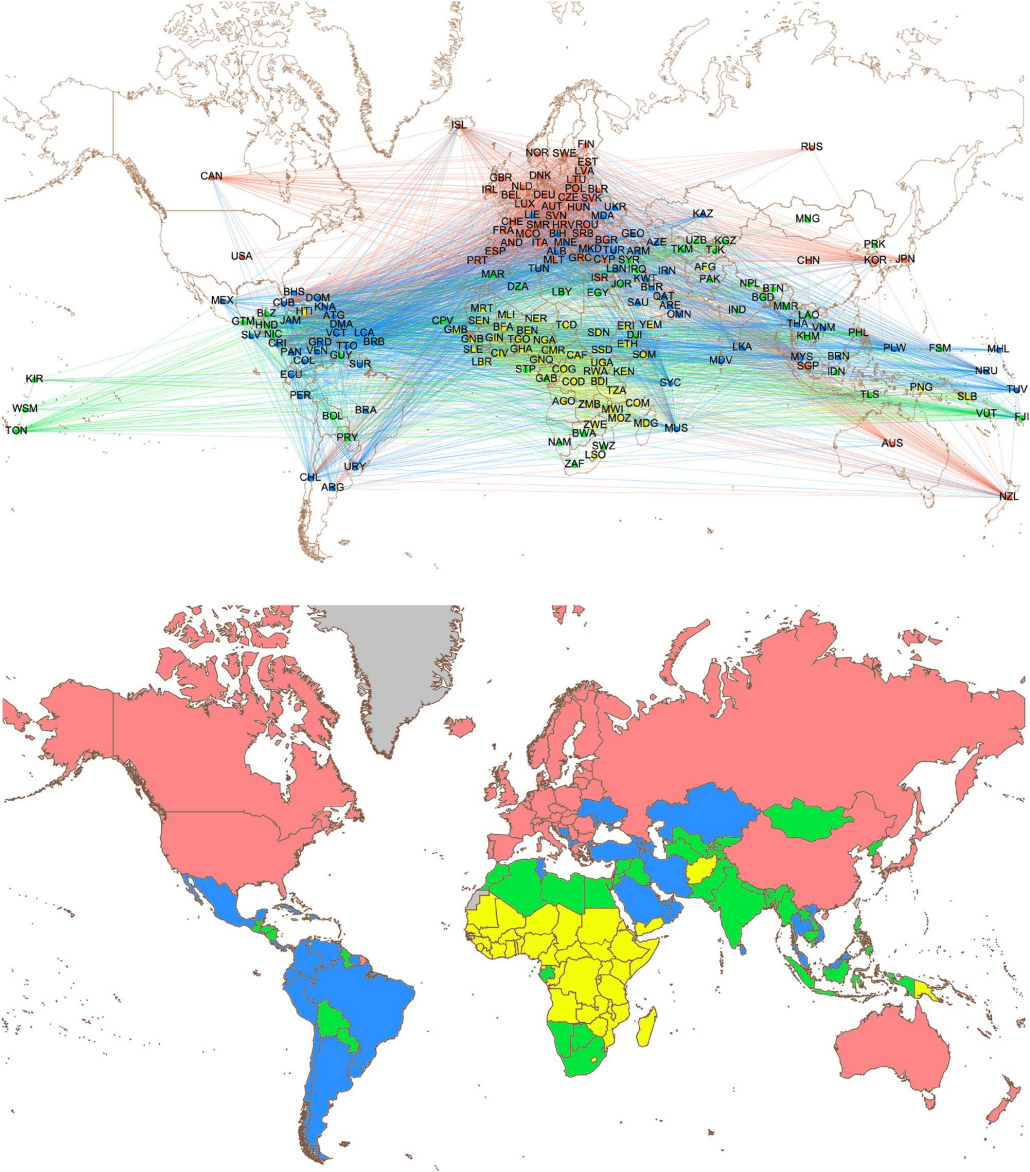
WDI network and communities. Upper panel: 2018 WDI complex network, with nodes representing UN countries and edge weights obtained from their pairwise Pearson correlation; only links with weight exceeding 0.65 are displayed. Countries on the map are labelled by their ISO 3166-1 alpha-3 codes^61^. Colors of nodes and intra-community edges are determined by the community to which they belong (red for community I, blue for II, green for III, yellow for IV), while edges connecting nodes from different communities take their color from the first node in alphabetical order. Lower panel: Geographical distribution of the four communities of the WDI complex network. States and territories not considered in the analysis are colored in grey. Maps are generated with the “Map of Countries” and “GeoLayout” plugins of Gephi 0.9.2^62^.

**Figure 2:**
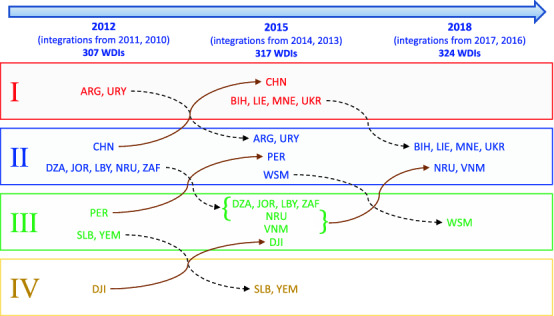
Variations in the membership of the four WDI communities from the 2012 network to the 2018 network.

The present work is organized as follows. In "Results" section, we shall focus on the main outcomes of the proposed approach: (a) a complex network of UN states with a community structure unveiling their similarity; (b) straightforward and validated procedures, based on the analysis of ranked index distributions inside and across network communities, to reconsider country performances in rankings in view of their development status. Implications of our findings and added value of the developed methodology will be addressed in "Discussion" section, while all steps of model development, from data construction to validation of development communities, will be discussed in "Methods" section; further details on our results will be provided in the Supplementary Information.

## Results

In this section, we present the main findings of our work. First, we discuss the most relevant features of the constructed UN development network and the structure of its partition in communities. Then, we quantitatively compare such partition with two established country groupings employed by the United Nations and the World Bank. Finally, we develop an equity-oriented reinterpretation of rankings, based on network communities. We investigate the possibility to apply our pipeline to five different rankings, chosen as representatives of different aspects of development, namely E-government Development Index (EGDI) 2018^63^, Environmental Performance Index (EPI) 2018^64,65^, Global Gender Gap Index (GGGI) 2020^66^, Healthcare Access and Quality Index (HAQI) 2016^67^, and SDG Global Index Score (SDGGIS) 2019^68^. We also introduce rating systems, determined by the index distributions across the network communities, and discuss their application to the considered rankings.

### WDI network communities

Using World Development Indicator (WDI) values^17^, we design a complex network model to capture similarities and differences among the 193 UN Member States (UNMS), listed in Supplementary Tab. S1 together with their current officially assigned ISO 3166-1 alpha-3 codes^61^. The choice of working with WDIs to construct our network is determined by the need for a representation of development as multidimensional as possible. Actually, WDI values include a wide variety of data; in our work, we consider indicators from the categories Environment, Economic Policy and Debt, Education, Financial Sector, Gender, Health, Infrastructure, Private Sector and Trade, Social Protection and Labor, covering essentially all aspects of a country’s condition. We will show in the following how such a multifaceted representation provides advantages in the interpretation of international rankings. In our network, each node corresponds to a UN country, which can be compared to the others through a proper similarity metrics. The network structure is therefore complete, namely each nation is connected to all the others by an edge. These edges are weighted with a measure of similarity between the countries they connect, based on pairwise Pearson correlation between their WDI values. In the present study, we consider networks based on the 2018, 2015 and 2012 WDI data, with missing entries integrated by the values from the 2 years before each reference year. Technical aspects and details about indicator selection, data filling and network construction are discussed in "Methods" section and in Supplementary Sec. S2. The upper panel of Fig. 1 shows the most relevant connections between countries in the 2018 WDI network, which therefore contains integrations from 2017 and 2016.

Hierarchical community detection is performed by means of two independent algorithms, explored in wide ranges of their parameter spaces (see Supplementary Sec. S5). A robust subdivision of the WDI network of UNMS in four communities (I, II, III, IV), represented in the lower panel of Fig. 1 for the 2018 case, emerges from this analysis. Interestingly, countries belonging to the the same community generally share geographical or economic proximity. Comparison with the partitions determined by the UN development groups^58^ and the World Bank income groups^59^ indicates a descending ordering from I to IV in terms of average development level (see Supplementary Sec. S6 for details); this observation justifies the term *development communities*, that will be employed henceforth. The relation between these three partitions of UN countries can be formalized by computing an index of similarity between them. A possible choice is the Normalized Mutual Information (NMI)^69^, a quantity defined in [0, 1], with 1 corresponding to maximal similarity. Using this metrics, an interesting result emerges: WDI network communities are more similar to both UN development groups ($\mathrm {NMI}=0.47$) and World Bank income groups ($\mathrm {NMI}=0.45$), than the two established country groupings are between them ($\mathrm {NMI}=0.36$). We can interpret this result as an effect of the multidimensionality of the data on which the WDI network is based, leading to a partition in communities that successfully interpolates between groupings that focus on different and more specific dimensions of development.

Finally, we observe that the partition of the network into development communities features a noticeable stability in time. Actually, the distribution of UNMS across development communities undergoes few modifications from the 2012 to the 2018 network, as shown in the diagram in Fig. 2. These results provide the basis of a new equity-oriented interpretation of international rankings, discussed in the following, that can help policy-makers and stakeholders define their agenda in view of the 2030 target date of UN Sustainable Development Goals.

### Rethinking rankings in the framework of development communities

**Figure 3:**
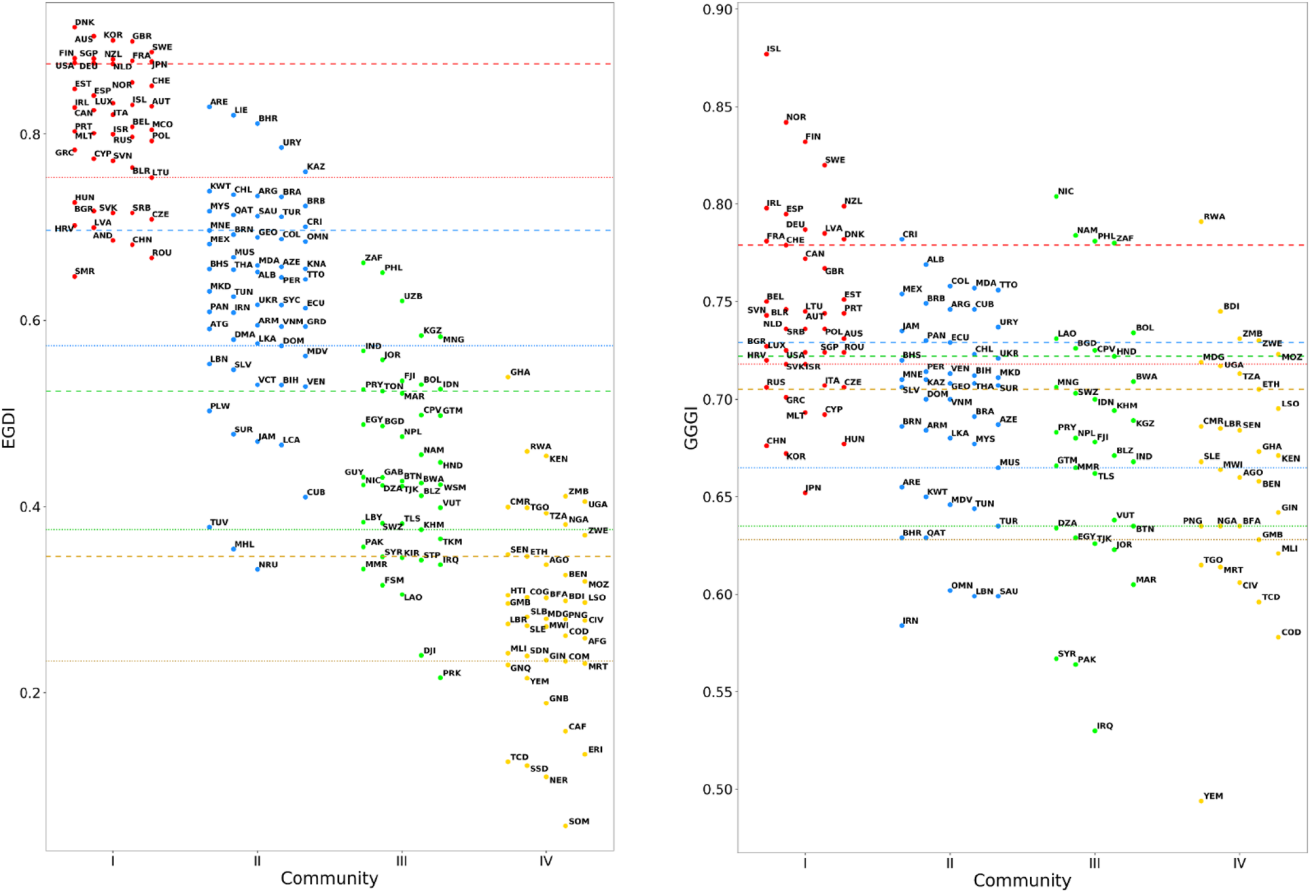
Distribution of EGDI (left panel) and GGGI (right panel) indexes of UN countries, separated in communities I (red), II (blue), III (green) and IV (yellow). Dashed and dotted lines of the same color as communities represent the 25th and 75th percentile, respectively, of the associated community distributions. Notice that, while the horizontal grouping of points is related to the community membership of the countries they represent, the specific abscissa of a point is not meaningful. Higher-resolution versions of the above plots are reported in Supplementary Sec. S7.

The communities identified from the network analysis provide a tool to group countries by development similarity, and also a way to reinterpret their positions in international rankings. Assuming that countries in each given WDI network community are characterized by essentially homogeneous development levels, it is natural to expect that, given the ranking of an index related to development, the ranked values would tend to cluster together inside each community and separate from the values of countries in other communities. Such a picture would also help to identify countries whose performance goes beyond the expectations based on their development status and countries that have the potential to reach their community peers in the ranking by increasing their efforts. However, as shown in Fig. 3, representing the distributions of EGDI (left panel) and GGGI (right panel) in the development communities, the validity of the aforementioned assumption may vary from one index to the other. In the two panels of Fig. 3, each country corresponds to a point whose vertical coordinate represents the value of the considered index, while the position with respect to the horizontal axis is determined by its community membership. The distributions highlight a qualitative difference between the two rankings: in the case of EGDI, community distributions are evenly spaced, partially overlapping, and following the same ordering as expected from the development hierarchy; on the other hand, as concerns the GGGI index, the community distributions are largely overlapped, with only community I slightly separated from the others. Therefore, in a case similar to GGGI, a community-based evaluation of country performance would not provide reliable information. The qualitative difference between the two cases is motivated by the fact that the considered indexes are not equally related with development: while EGDI aims at providing an evaluation of a country’s E-government capabilities (in which, intuitively, developed countries are advantaged), GGGI measures gender gap with respect to a standard that varies state by state, depending on the political, economic, health and education condition.

**Figure 4:**
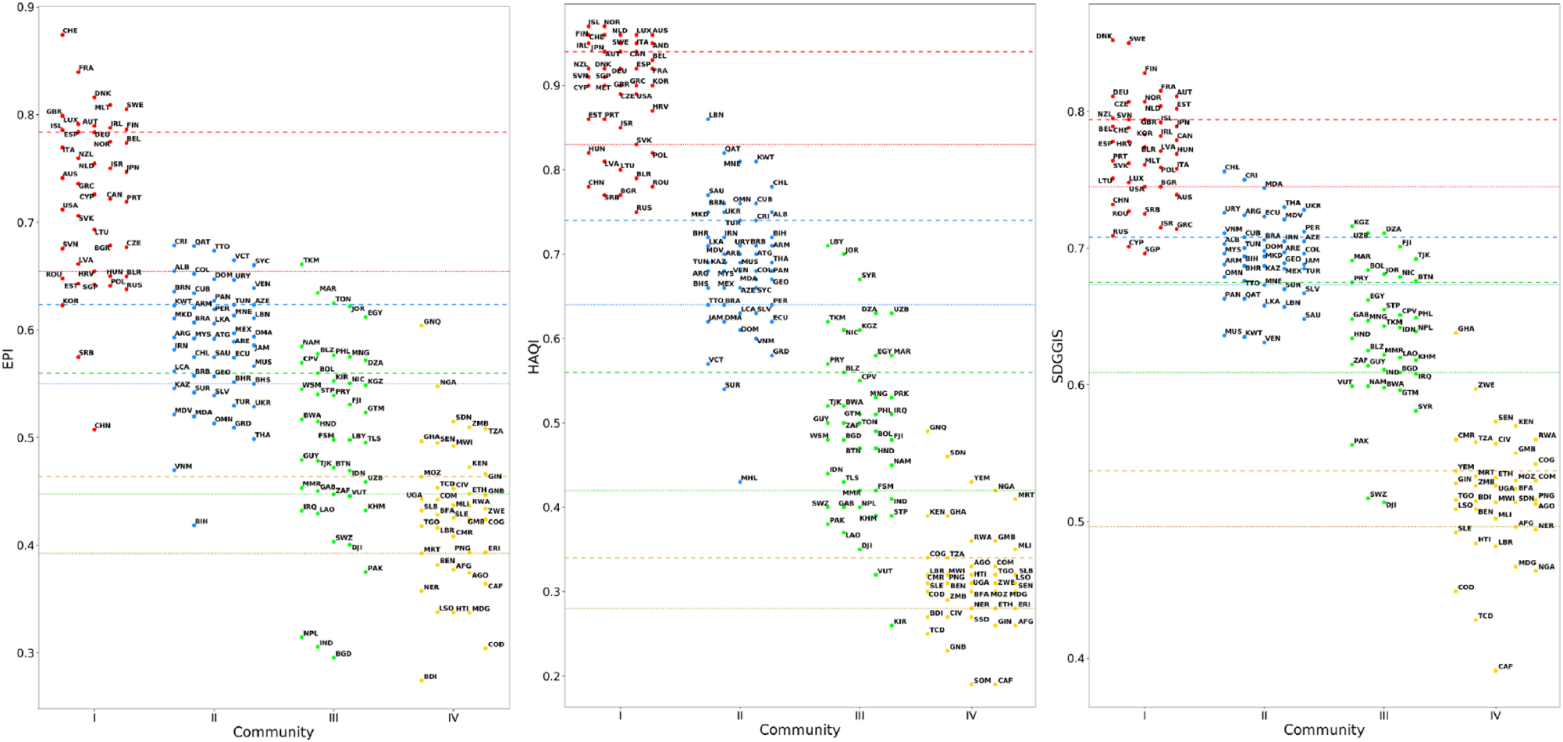
Distribution of EPI (left panel), HAQI (central panel) and SDGGIS (right panel) indexes of UN countries, separated in communities I (red), II (blue), III (green) and IV (yellow). Dashed and dotted lines of the same color as communities represent the 25th and 75th percentile, respectively, of the associated community distributions. While the horizontal grouping of points is related to the community membership of the countries they represent, the specific abscissa of a point is not meaningful. Higher-resolution versions of the above plots are reported in Supplementary Sec. S7.

In order to quantify the relatedness between the performance of countries in a ranking and their community membership, we introduce a quality factor *R*, called the *resolution ratio*, that is as larger as the community index distributions are more separated. The resolution ratio, whose definition is given in "Methods" section, is much larger than one when the community index distributions have a limited overlap with each other, and much smaller than one when the overlap is practically full. In the intermediate case $R\simeq 1$ the separation between the mean values of neighboring community distributions is similar to the typical variation of the index within each community. The value $R=1$ can be assumed as a threshold to distinguish cases in which the evaluation of country performances based on development communities is either meaningful or not. Table 1 summarizes the resolution ratios obtained for the five indexes considered in our analysis. As expected from the qualitative considerations on Fig. 3, the development communities are well resolved with respect to EGDI and insufficiently separated in the case of GGGI. The highest ratios are obtained for HAQI and SDGGIS, reflecting the strong relation between these indexes and development as a whole, while the result for EPI is lower, although still considerably larger than 1. The distributions of EPI, HAQI and SDGGIS are reported in Fig. 4.

### Evaluation of country performances in rankings

In the case $R>1$, reasonable predictions on the performance of countries in the considered ranking may be done on the basis of community membership, and deviations from the expected behavior can be critically evaluated. The attention can be focused on countries whose performance in a given ranking is either above or below expectations determined by the results of countries in the same community and in other communities. Specifically, we can identify *top-of-the-class* countries in the ranking as those whose ranked index values fall, at the same time

- above the 75th percentile of the community they belong,
- above the 25th percentile of at least one more developed community.

Following a similar criterion, we define as *room-for-improvement* countries the ones whose index values are located both

- below the 25th percentile of the community they belong,
- below the 75th percentile of at least one less developed community.

Top-of-the-class countries can be seen as reference cases that could inspire the action of similar states to improve their status in the ranking. To further quantify the mismatch between the performances of such states and expectations based on their community membership, we award a top-of-the-class country with a symbol “$\uparrow$” for each 25th percentile of a more developed community that is overcome by its index value. On the other hand, room-for-improvement countries can be interpreted as states which have the potential, in terms of development, to achieve better results in the considered ranking and close the gap with similar countries. In this case, we mark a country with a symbol “$*$” each time the index value falls below the 75th percentile of a less developed community.

States with the highest indexes in community I and with the lowest indexes in community IV are not covered by the above definitions, due to the lack of more and less developed communities, respectively. Therefore, for a comprehensive mapping of remarkable performances in ranking, we introduce the categories of *benchmark* and *trailing* countries. Benchmark countries are states belonging to community I, characterized by an index value above the 75th community percentile; they play a crucial role in policy design, being regarded as best-practice by the rest of the world. Trailing countries belong to community IV, with their index values falling below the 25th community percentile; these states are noteworthy because they could need specific support to improve their condition in the development areas related to the ranking. In the following, referring to Figs. 3 and 4, we shall assess country performances in each community for the rankings with $R>1$ analyzed in this work.

**Table 1:** Resolution ratio of the rankings determined by the considered indexes, with respect to the partition of the UN member states into the four development communities.

	EGDI	EPI	GGGI	HAQI	SDGGIS
Resolution ratio of WDI communities	3.76	2.29	0.32	7.51	5.26

#### E-government Development Index 2018

The EGDI measures the effort and ability of all the 193 UNMS in using ICTs to provide public services at the national level, through the assessment of three key-aspects: the development of telecommunication infrastructure, the capacity-building in human capital and the quality of online service delivery^63^. In the case of 2018 EGDI, the distributions related to neighboring communities in the left panel of Fig. 3 partially overlap. Performances of UN states can be assessed from their development status according to the aforementioned criterion, with results summarized as follows:

- *Community I* (median: 0.814). *Benchmark*: Denmark, Australia, Rep. Korea, United Kingdom, Sweden, Finland, Singapore, New Zealand, France, Japan, United States, Germany. *Room-for-improvement*: Andorra ($*$), China ($*$), Romania ($*$), San Marino ($*$).
- *Community II* (median: 0.645). *Top-of-the-class*: United Arab Emirates ($$\uparrow$$), Liechtenstein ($$\uparrow$$), Bahrain ($$\uparrow$$), Uruguay ($$\uparrow$$), Kazakhstan ($$\uparrow$$). *Room-for-improvement*: Palau ($$*$$), Suriname ($$*$$), Jamaica ($$*$$), Saint Lucia ($$*$$), Cuba ($$*$$), Tuvalu ($$*$$), Marshall Islands ($$*$$), Nauru ($$**$$).
- *Community III* (median: 0.427). *Top-of-the-class*: South Africa ($\uparrow$), Philippines ($\uparrow$), Uzbekistan ($\uparrow$), Kyrgyz Republic ($\uparrow$), Mongolia ($\uparrow$). *Room-for-improvement*: Syrian Arab Rep. ($*$), Kiribati ($*$), Sao Tome and Principe ($*$), Iraq ($*$), Myanmar ($*$), Fed. Sts. Micronesia ($*$), Lao PDR ($*$), Djibouti ($*$), Dem. People’s Rep. Korea ($*$).
- *Community IV* (median: 0.280). *Top-of-the-class*: Ghana ($\uparrow$), Rwanda ($\uparrow$), Kenya ($\uparrow$), Zambia ($\uparrow$), Uganda ($\uparrow$), Cameroon ($\uparrow$), Togo ($\uparrow$), Tanzania ($\uparrow$), Nigeria ($\uparrow$). *Trailing*: Mauritania, Equatorial Guinea, Rep. Yemen, Guinea-Bissau, Central African Republic, Eritrea, Chad, South Sudan, Niger, Somalia.

The assessment identifies 19 top-of-the-class and 21 room-for-improvement countries, corresponding to the 9.8% and 10.9% of ranked countries, respectively.

#### Environmental Performance Index 2018

The EPI^64,65^ is computed from a set of indicators related to different issues, covering environmental health and ecosystem vitality, and measures the achievement level of the established environmental policy goals of countries. The 2018 ranking reports values for 179 UNMS, providing the following ratings:

- *Community I* (median: 0.736). *Benchmark*: Switzerland, France, Denmark, Malta, Sweden, United Kingdom, Luxembourg, Austria, Ireland, Finland, Iceland. *Room-for-improvement*: Rep. Korea ($$*$$), Serbia ($$*$$), China ($$**$$).
- *Community II* (median: 0.593). *Top-of-the-class*: Costa Rica ($$\uparrow$$), Qatar ($$\uparrow$$), Trinidad and Tobago ($$\uparrow$$), Saint Vincent and the Grenadines ($$\uparrow$$), Seychelles ($$\uparrow$$), Albania ($$\uparrow$$). *Room-for-improvement*: Kazakhstan ($$*$$), Suriname ($$*$$), El Salvador ($$*$$), Turkey ($$*$$), Ukraine ($$*$$), Maldives ($$*$$), Moldova ($$*$$), Oman ($$*$$), Grenada ($$*$$), Thailand ($$*$$), Vietnam ($$*$$), Bosnia and Herzegovina ($$**$$).
- *Community III* (median: 0.515). *Top-of-the-class*: Turkmenistan ($\uparrow \uparrow$), Morocco ($\uparrow$), Tonga ($\uparrow$), Jordan ($\uparrow$), Arab Rep. Egypt ($\uparrow$), Namibia ($\uparrow$), Belize ($\uparrow$), Philippines ($\uparrow$), Mongolia ($\uparrow$), Algeria ($\uparrow$), Cabo Verde ($\uparrow$). *Room-for-improvement*: Vanuatu ($*$), Cambodia ($*$), Iraq ($*$), Lao PDR ($*$), Eswatini ($*$), Djibouti ($*$), Pakistan ($*$), Nepal ($*$), India ($*$), Bangladesh ($*$).
- *Community IV* (median: 0.432). *Top-of-the-class*: Equatorial Guinea ($\uparrow \uparrow$), Nigeria ($\uparrow$), Sudan ($\uparrow$), Zambia ($\uparrow$), Tanzania ($\uparrow$), Ghana ($\uparrow$), Senegal ($\uparrow$), Malawi ($\uparrow$), Kenya ($\uparrow$), Guinea ($\uparrow$). *Trailing*: Benin, Afghanistan, Angola, Central African Republic, Niger, Lesotho, Haiti, Madagascar, Dem. Rep. Congo, Burundi.

The assessment identifies 27 top-of-the-class and 25 room-for-improvement countries, corresponding to the 15.1% and 14.0% of ranked countries, respectively.

#### Healthcare Access and Quality Index 2016

The HAQI, compiled by the GBD 2016 Healthcare Access and Quality collaboration^67^, evaluates the health services based on death rates from 32 causes for which death could be avoided by timely and effective medical care. The 2016 index is available for 186 UNMS and provides the following performance ratings:

- *Community I* (median: 0.905). *Benchmark*: Iceland, Norway, Netherlands, Luxembourg, Australia, Finland, Switzerland, Sweden, Italy, Andorra, Ireland. *Room-for-improvement*: none.
- *Community II* (median: 0.690). *Top-of-the-class*: Lebanon ($\uparrow$). *Room-for-improvement*: Suriname ($*$), Marshall Islands ($*$).
- *Community III* (median: 0.500). *Top-of-the-class*: Libya ($\uparrow$), Jordan ($\uparrow$), Syrian Arab Republic ($\uparrow$). *Room-for-improvement*: Vanuatu ($*$), Kiribati ($*$).
- *Community IV* (median: 0.315). *Top-of-the-class*: Equatorial Guinea ($\uparrow$), Sudan ($\uparrow$), Rep. Yemen ($\uparrow$). *Trailing*: Burundi, Cote d’Ivoire, South Sudan, Guinea, Afghanistan, Chad, Guinea-Bissau, Somalia, Central African Republic.

The assessment identifies 7 top-of-the-class and 4 room-for-improvement countries, corresponding to the 3.8% and 2.2% of ranked countries, respectively.

#### SDG Global Index Score 2019

The SDGGIS is presented in the annual Sustainable Development Report^68^ and quantifies the overall level of achievement of Sustainable Development Goals. The index for 2019 is available for 162 UNMS. The performance of countries is evaluated as follows:

- *Community I* (median: 0.778). *Benchmark*: Denmark, Sweden, Finland, France, Austria, Germany, Czech Republic, Norway, Netherlands, Estonia, New Zealand. *Room-for-improvement*: Cyprus ($*$), Singapore ($*$).
- *Community II* (median: 0.694). *Top-of-the-class*: Chile ($\uparrow$), Costa Rica ($\uparrow$). *Room-for-improvement*: Suriname ($*$), El Salvador ($*$), Panama ($*$), Qatar ($*$), Sri Lanka ($*$), Lebanon ($*$), Saudi Arabia ($*$), Mauritius ($*$), Kuwait ($*$), RB Venezuela ($*$).
- *Community III* (median: 0.641) *Top-of-the-class*: Kyrgyz Republic ($\uparrow$), Uzbekistan ($\uparrow$), Algeria ($\uparrow$), Fiji ($\uparrow$), Tajikistan ($\uparrow$), Morocco ($\uparrow$), Bolivia ($\uparrow$), Jordan ($\uparrow$), Nicaragua ($\uparrow$), Bhutan ($\uparrow$). *Room-for-improvement*: Eswatini ($*$), Djibouti ($*$).
- *Community IV* (median: 0.520) *Top-of-the-class*: Ghana ($\uparrow$). *Trailing*: Niger, Sierra Leone, Haiti, Liberia, Madagascar, Nigeria, Dem. Rep. Congo, Chad, Central African Republic.

The assessment identifies 13 top-of-the-class and 14 room-for-improvement countries, corresponding to the 8.0% and 8.6% of ranked countries, respectively.

#### A community-based rating scheme

To complete the analysis of country performances in rankings in view of their development status, we introduce a rating criterion, based on crossing information on the position of each UNMS in the overall ranking with its membership to a community of the WDI network. This framework, whose results in the case of EGDI are illustrated in Table 2, is made of the following steps:

1. The range of the ranked index is partitioned in the quartiles ($\mathrm {Q}_1,\mathrm {Q}_2,\mathrm {Q}_3,\mathrm {Q}_4$), in descending order of values. Each country receives a primary rating, consisting in a capital letter (A, B, C, D), according to the quartile in which its index value falls.
2. Countries in a given quartile are assigned as many small-case versions of the primary-rating letter as the number of more developed communities with representatives in the same quartile.

The secondary rating enforces equity by rewarding states that reach the same quartile as at least one member of a more developed community. Ratings with respect to other indexes with $R>1$ are reported in Supplementary Tabs. S9–S11.

**Table 2:** Classification of countries based on the quartiles ($\mathrm {Q}_1,\mathrm {Q}_2,\mathrm {Q}_3,\mathrm {Q}_4$) of the overall EGDI distribution and on the development community membership.

	I	II	III	IV
$\mathbf{Q} _{{1}}$	**A** DNK, AUS, KOR, GBR, SWE, FIN, SGP, NZL, FRA, JPN, USA, DEU, NLD, NOR, CHE, EST, ESP, LUX, ISL, AUT, IRL, CAN, ITA, BEL, MCO, PRT, MLT, ISR, RUS, POL, GRC, CYP, SVN, BLR, LTU, HUN, BGR	**Aa** ARE, LIE, BHR, URY, KAZ, KWT, CHL, ARG, BRA, BRB, MYS		
$\mathbf{Q} _{{2}}$	**B** SVK, SRB, CZE, HRV, LVA, AND, CHN, ROU, SMR	**Bb** QAT, SAU, TUR, CRI, MNE, BRN, GEO, COL, OMN, MEX, MUS, MDA, AZE, KNA, BHS, THA, ALB, PER, TTO, MKD, TUN, UKR, SYC, ECU, PAN, IRN, ARM, VNM, GRD, ATG, DMA, LKA, DOM	**Bbb** ZAF, PHL, UZB, KGZ, MNG, IND	
$\mathbf{Q} _{{3}}$		**C** MDV, LBN, SLV, VCT, BIH, VEN, PLW, SUR, JAM, LCA, CUB, TUV	**Cc** JOR, FJI, BOL, IDN, PRY, TON, MAR, CPV, GTM, EGY, BGD, NPL, NAM, HND, GUY, GAB, BTN, BWA, WSM, NIC, DZA, TJK, BLZ, VUT, LBY, SWZ, TLS	**Ccc** GHA, RWA, KEN, ZMB, UGA, CMR, TGO, TZA, NGA
$\mathbf{Q} _{{4}}$		**D** MHL, NRU	**Dd** KHM, TKM, PAK, SYR, KIR, STP, IRQ, MMR, FSM, LAO, DJI, PRK	**Ddd** ZWE, SEN, ETH, AGO, BEN, MOZ, HTI, COG, BFA, BDI, LSO, GMB, SLB, MDG, PNG, CIV, LBR, SLE, MWI, COD, AFG, MLI, SDN, GIN, COM, MRT, GNQ, YEM, GNB, CAF, ERI, TCD, SSD, NER, SOM

## Discussion

Let us summarize the main findings of the work presented in this paper: the emergence of a robust community structure in the complex network obtained from WDI values; the quantification through a quality factor of how much the distributions of a given ranked index in different communities are separated; the introduction of a straightforward method, based on WDI communities, to reinterpret rankings.

The complex network is constructed, following the proximity principle^38^, starting from correlations between sets of WDIs pertaining to different countries. The resulting network provides information on the development both on a state-specific level and on a collective level. Information on how each state is correlated to the rest of the network is synthesized in Supplementary Fig. S4 and Table S8. From the results therein, one can deduce that some countries are characterized by generally weak correlations, and therefore low affinity with other states. In particular, the following countries have an average correlation smaller than 0.25: India (0.2395), Dem. Rep. Congo (0.2293), Chad (0.2216), China (0.2090), Somalia (0.1674), South Sudan (0.1671), United States (0.1334). Three of them are placed in extremal positions of the whole development spectrum, thus often featuring as least-correlated states (see third column of Supplementary Table S8), and represent almost all the entries of such category: the United States are least-correlated for 85 countries, Somalia for 66, South Sudan for 30. Another interesting case is represented by China, due to its peculiarity, as confirmed by not only the average correlation, but even its maximal value (namely, 0.5719 with Russian Federation), which represents the only maximal correlation below 0.6 in the network. In general, maximal similarity relations follow geographical proximity: in 43% cases, the most correlated country is a state that shares a land border. Interesting exceptions to this rule are, e.g., the reciprocal maximal correlation between Canada and Australia, and the fact that the maximally correlated states to Japan and New Zealand are Germany and Iceland, respectively. Future research will be devoted to scrutinize the impact of the indicator selection procedure on both UNMS mutual similarities and their community membership. Knowledge of these mechanisms can provide a relevant instrument to modulate policy design in different development areas. A further perspective is represented by the use of a set of optimized indicator combinations, such as the cluster-driven composite indicators proposed in Ref.^35^, to construct the network. Such a choice, which goes beyond the scope of this work, would constitute a refined and controllable alternative to the indicator selection process described in "Data collection and preprocessing" section.

As for the value of our model on a collective level, we remark that the partition of world countries based on the WDI network largely fulfills the requirements for a good classification scheme, proposed by the Expert Group on International Economic and Social Classifications^70,71^, including determination of exhaustive and mutually exclusive categories, comparability with related standard classifications, stability in time, solid data-driven foundations and explanations, and a reasonable number of groups, reflecting reality of the field. In addition, the similarity patterns found by community detection in the WDI network are neither trivial nor redundant with those obtained from traditional approaches, e.g. geographical criteria or methods employed by UN and World Bank, based on country development and income, respectively. Nonetheless, we find a general coherence of our results with the aforementioned grouping procedures. Specifically, by interpreting the results of the NMI between the WDI communities, the UN development groups and the World Bank income groups, we are able to determine that the partition emerging from our model represents an interpolation between the two established groupings. The idea of grouping countries starting from a given set of indicators has already been considered in literature, where hierarchical clustering based on a small number of indicators was applied to problems concerning the technological gap^72^ and the relation between innovation and competitiveness^73^. It is worth noticing that the use of hierarchical clustering to partition our WDI network leads to very uneven country groups, in which the less correlated states (see discussion above) tend to be isolated. Complex network models have also been employed to characterize countries and their mutual relations, mainly in studies with a socio-economic and political background^48,56^. These works investigate the existence of possible hierarchies, unveiled by centrality measures^57^, as well as influences of one state on another^74,75^. To the best of our knowledge, no previous study has focused on the reinterpretation of rankings through the analysis of mutual similarity relations between countries in a complex network framework. Our model provides an immediate and versatile procedure to evaluate the discrepancy between a country’s performance in a ranking and its development condition. This assessment is based on comparison both inside a community and with other communities, namely with different development levels.

Comparing the distributions of ranked indexes across the different communities, as well as the numbers of ($\uparrow$) and ($*$) points awarded in each ranking, we observe that our evaluation method can feature varying sensitivities, depending on the value of the resolution ratio *R*. In particular, when the index results from indicators that are strongly related to the selected WDIs, the ranking is characterized by a large *R* and its distributions within different communities tend to separate from each other, with only few states performing differently from what expected on the basis of their development community membership. When the redundancy between the ranked index and WDIs is lower, the overlap between community distributions is larger, and a higher number of states receive a mark, either positive or negative. Finally, marks become pointless in the case $R<1$, when community distributions are mostly overlapped with each other. It is worth stressing that the assignment of ($\uparrow$) and ($*$) ratings allows the comparison of countries within the same ranking, and cannot be used to compare the performance of a given country in different rankings, since higher redundancies between the index and the WDIs generally yield a smaller number of both awards ($\uparrow$) and penalties ($*$). In a ranking characterized by a high *R* with respect to the WDI network partition, the attribution of a negative rating should thus be considered much more worrying than if the same penalty were attributed in a less WDI-related ranking. The discussed regimes are also observed in the community-based rating, described in "Evaluation of country performances in rankings" section. For strongly WDI-related rankings, index values of countries belonging to communities I, II, III and IV are concentrated in the quartiles $\mathrm {Q}_1$, $\mathrm {Q}_2$, $\mathrm {Q}_3$ and $\mathrm {Q}_4$, respectively. Instead, the spreading of communities across quartiles is much more pronounced in the case of weakly WDI-related indexes, entailing the assignment of a larger number of secondary ratings.

**Table 3:** Comparison between resolution ratios of ranked indexes with respect to the partitions determined by WDI communities, UN development groups, and World Bank income groups. Only results for rankings with significantly separated community index distributions ($R>1$) are reported.

	EGDI	EPI	HAQI	SDGGIS
WDI communities	3.76	2.29	7.51	5.26
UN development groups	1.86	1.57	2.82	2.33
World Bank income groups	2.40	1.89	3.16	2.04

A natural question arises as to why introducing a network-based partition to reinterpret rankings, instead of using established groupings such as UN development groups and World Bank income groups. The reasons of the first choice are both conceptual and phenomenological. From a conceptual point of view, WDI communities emerge from a network based on several sectors of the development spectrum, and are obtained through numerical criteria in an unsupervised way, without bias on the arrangement of countries in groups. On the other hand, World Bank income groups have been set focusing on a narrower aspect of development, while UN development groups and other groupings are based on a small number of indicators, chosen and aggregated according to criteria that can suffer from different kinds of bias and variation in time^60^. From a phenomenological point of view, the evaluation of country performance in a ranking, especially when deviations from the expected result occur, is as reliable as the partition is related to the ranked index values^71^. In Table 3 we compare the resolution ratios of the rankings with respect to the aforementioned partitions, focusing on the cases in which the proposed rating scheme is meaningful ($R>1$). It is evident that WDI network communities provide the highest resolution ratio for *all* the indexes, thus representing the sharpest way of the three to identify discrepancies between rankings and development. We argue that the improved *R* for all the rankings is due to both the multidimensionality of WDI dataset and the absence of possible biases in obtaining the partition.

The proposed methodology highlights the different facets of a country’s condition, which are often the result of the actions undertaken and priorities established by its government. Development communities and rating criteria define a rigorous, transparent and reproducible procedure, that can provide a basis for policy design. The aim of our analysis is not to pass judgments, that could be simplistic, on state performances, but rather to highlight, for each country, strengths and unexpressed potentialities. This kind of information can be crucial in supporting the definition of adequate and promising development trajectories. In particular, top-of-the-class states could target a general improvement of their WDIs, starting from the specific fields in which they excel. On the other hand, countries labeled as room-for-improvement in a specific ranking have concrete growth perspective, since they are provided with the necessary resources to address policies towards a better result in that field. This information, emerging in our model, can have a pivotal value in development strategy planning, as well as to assess the effects of measures already adopted, e.g. the public policies undertaken over the years to strengthen ICT-related mechanisms for public benefit in Ghana, Rwanda and Uganda^41,63^, recognized in our scheme as community IV top-of-the-class countries in the EGDI ranking.

## Methods

**Figure 5:**
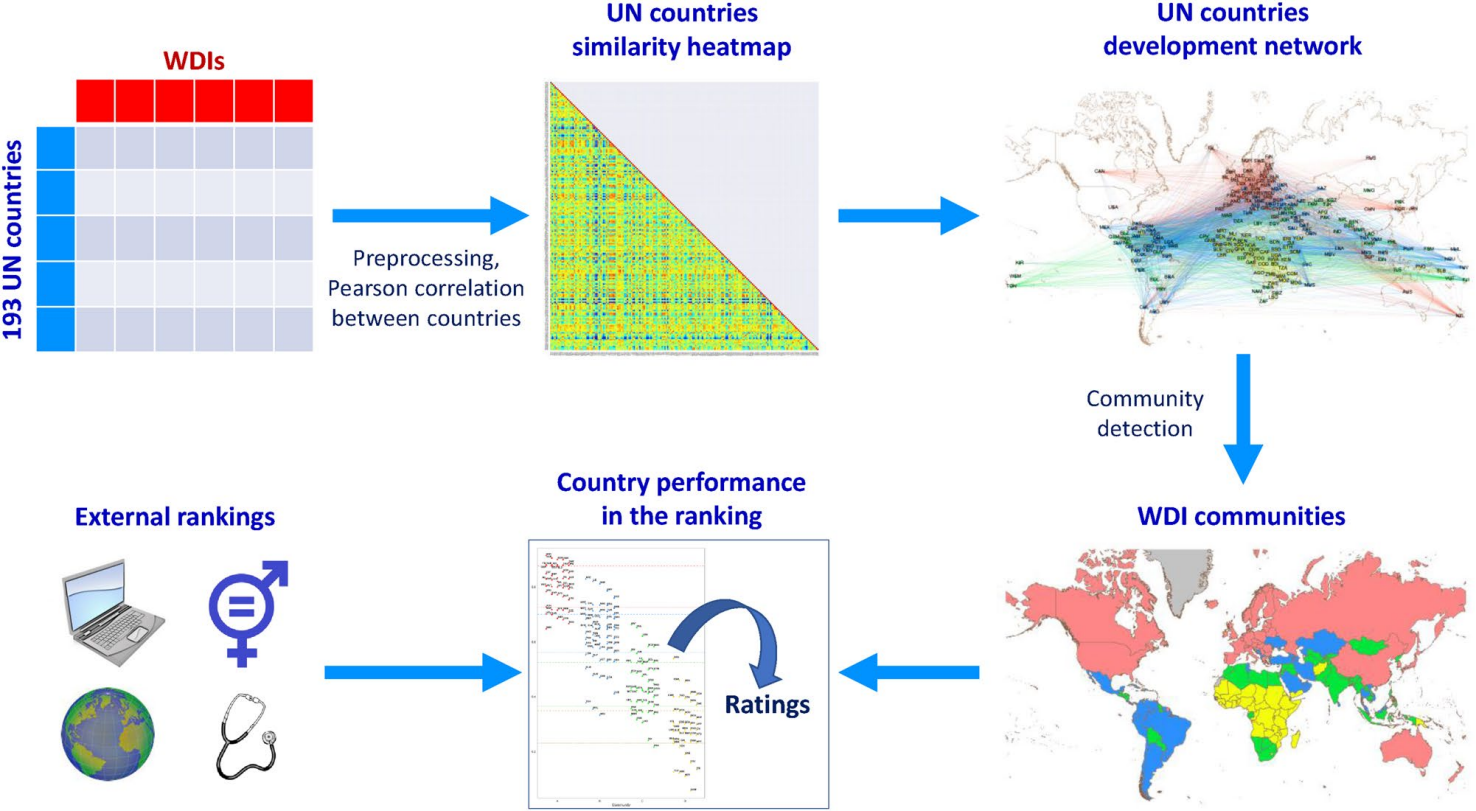
Scheme representing the pipeline of the analysis. A portion of WDI database^17,76^ is selected according to availability criteria. A complex network describing similarity of countries is obtained from pairwise Pearson correlations between their WDIs, after rescaling each WDI in [0, 1]. Community detection results are compared with index rankings with resolution ratio $R>1$, in order to evaluate performances of countries according to their development status. Maps are generated with the “Map of Countries” and “GeoLayout” plugins of Gephi 0.9.2^62^.

In the following, we shall focus on the methods employed to construct and analyze a complex network in which UN countries, representing nodes, are mutually connected with increasing strength, according to their similarity. Specifically, a community detection based on WDIs is performed and used as a touchstone to interpret various country rankings. A scheme representing the pipeline of this study is displayed in Fig. 5.

### Data collection and preprocessing

#### World Development Indicators

The database on which the network is built is represented by the World Development Indicators (WDIs)^17^, “a compilation of relevant, high-quality, and internationally comparable statistics about global development and the fight against poverty”, containing time-series indicators, going back to 1960 in the best case, for 217 economies (referred to both UN and non-UN countries) and more than 40 economic or geographical country groups. We choose to focus on the 193 countries belonging to the UN^77^, listed in Supplementary Table S1 along with their official ISO 3166-1 alpha-3 abbreviation codes.

The bulk file used for the analysis was released on June 11, 2010 and last updated on March 18, 2020. The dataset records 1429 WDIs, but a large part of them is not available for all member states; in particular, the number of missing entries is subject to large fluctuations between one country and another. Data availability also depends on time: it increases with an approximately monotonous trend from 1960 to the period 2005–2016 (reaching a maximum in 2010), as a result of improvements in collection procedures, and drops in the subsequent years, since most recent results are still unrecorded. We choose, as our main task, to work on the 2018 indicators, as a compromise between indicator availability and freshness, borrowing missing entries from the 2017 part of the data or, if the latter were also missing, from the 2016 one. To perform our analysis, we select 324 indicators, according to a criterion of data availability, consistency and non-redundant information, described in detail in Supplementary Sec. S2. The missing entries in the 2018 dataset are partially filled with 9418 values of 2017 and 8768 values of 2016, eventually leaving 3098 not available entries in the resulting patched 2018 dataset. An analogous operation is performed for the datasets of 2015 (integrated with 2014 and 2013 data), 2012 (integrated with 2011 and 2010 data) and 2009 (integrated with 2008 and 2007 data). Supplementary Sec. S2 reports a comprehensive list of selected WDIs, together with countries with missing values for the patched 2018 dataset, and bar plots showing the availability of indicators for the patched datasets of 2018, 2015, and 2012. Fractions of missing data larger than $30\%$, reported in Supplementary Tab. S7, are observed in 2018 for microstates (Andorra, Dominica, Liechtenstein, Marshall Islands, Monaco, Nauru, Palau, San Marino, St. Kitts and Nevis, Tuvalu) and for other countries (Eritrea, Dem. People’s Rep. Korea) that do not disclose the values of many indicators.

The 324 selected indicators are finally normalized in order to compute Pearson correlation between different countries and develop the complex network model. More precisely, to mitigate the effect of outliers, the values exceeding the 99th percentile from above and the 1st percentile from below were replaced by the reference percentiles *before* performing the linear rescaling of the indicator in [0, 1].

#### E-government Development Index 2018

This index is available for all UN countries, and is defined in the range [0, 1], with 0 and 1 corresponding to the worst and best achievable performances, respectively. Therefore, no rescaling is required for the data shown in the left panel of Fig. 3.

#### Global Gender Gap Index 2020

This index is not available for the following countries: Afghanistan, Andorra, Antigua and Barbuda, Central African Republic, Comoros, Rep. Congo, Djibouti, Dominica, Equatorial Guinea, Eritrea, Gabon, Grenada, Guinea-Bissau, Guyana, Haiti, Kiribati, Dem. People’s Rep. Korea, Libya, Liechtenstein, Marshall Islands, Fed. Sts. Micronesia, Monaco, Nauru, Niger, Palau, Samoa, San Marino, Sao Tome and Principe, Seychelles, Solomon Islands, Somalia, South Sudan, St. Kitts and Nevis, St. Lucia, St. Vincent and the Grenadines, Sudan, Tonga, Turkmenistan, Tuvalu, Uzbekistan. GGGI ranges from 0 (largest possible gender gap) to 1 (smallest possible gender gap). Therefore, no rescaling is needed for the data shown in the right panel of Fig. 3.

#### Environmental Performance Index 2018

The UN countries for which this index is available do not include: Andorra, Dem. People’s Rep. Korea, Liechtenstein, Marshall Islands, Monaco, Nauru, Palau, San Marino, Somalia, South Sudan, St. Kitts and Nevis, Syrian Arab Republic, Tuvalu, Rep. Yemen. EPI ranges from 0 to 100, with the best achievable performance corresponding to the upper bound. In our work, we rescale the index in [0, 1], as reported in the left panel of Fig. 4, dividing its values by 100.

#### Healthcare Access and Quality Index 2016

The 2016 HAQI ranking does not include values for: Liechtenstein, Monaco, Nauru, Palau, San Marino, St. Kitts and Nevis, Tuvalu. The index attributes a score ranging from 0 (worst attainable performance) to 100 (best attainable performance), and is therefore rescaled dividing by 100 to obtain the data shown in the central panel of Fig. 4.

#### SDG Global Index Score 2019

The list of countries for which the 2019 SDG Global Index Score is not available, including mainly small island countries and other microstates, reads: Andorra, Antigua and Barbuda, The Bahamas, Barbados, Brunei Darussalam, Dominica, Equatorial Guinea, Eritrea, Grenada, Guinea-Bissau, Kiribati, Dem. People’s Rep. Korea, Libya, Liechtenstein, Marshall Islands, Fed. Sts. Micronesia, Monaco, Nauru, Palau, Samoa, San Marino, Seychelles, Solomon Islands, Somalia, South Sudan, St. Kitts and Nevis, St. Lucia, St. Vincent and the Grenadines, Timor-Leste, Tonga, Tuvalu. Since the index quantifies, in percent, the level of achievement of Sustainable Development Goals, its values are naturally rescaled in [0, 1], as reported in the right panel of Fig. 4, dividing them by 100.

### UN states development network

The WDIs, selected and rescaled according to the procedure described in Supplementary Sec. S2, are used to compute the pairwise Pearson correlations between countries. We use these correlations to construct a complex network made of 193 nodes, representing UN countries, linked by weighted edges, whose weight coincides with the pairwise Pearson correlation. The resulting network is made of $193\times 192/2=18{,}528$ links, being therefore complete. The choice to work with a complete network is motivated by a number of reasons. First, a network with a simpler topology, made of sparser connections, would require the definition of a threshold value for link weights, below which a pair of countries is considered disconnected. This procedure would lead us to different results according to the choice of the threshold, introducing arbitrariness in our analysis and loss of information; actually, weak and negative correlations between countries contribute to determine the network partition in communities. Moreover, an additional problem arising from the choice of trimming the network is the fact that the thresholding operation would act inhomogeneously on countries, erasing, in particular, the information on similarities and differences related to countries for which correlations are weak on average.

#### Community detection

The identification of communities must take into account the fact that the network is complete and weighted with both positive and negative edge weights. Thus, we choose two community detection algorithms that have recently been optimized in order to handle negative weights^46^, which is a rather uncommon feature: Spin Glass, based on statistical mechanics concepts^78,79^ and Leiden^80^. We perform for both algorithms hierarchical community detection by recursive partitioning, an approach already explored in Refs.^69,81,82^. In our multi-step procedure, subsequent detection is applied to partition the communities obtained at the previous stage, as long as an iteration condition is satisfied. Such condition is represented by the accordance between outputs of community detection for different runs of the algorithm at the considered step. At each step, communities are found using the algorithms of the *igraph* library^83^. Actually, each algorithm operates within a pipeline that is not entirely deterministic, yielding in principle different outputs when applied to the same network; however, the outcome of a robust community detection should be as independent as possible from randomness. To obtain reliable communities, we adopt the following criterion: the network is partitioned 100 times by one of the chosen algorithms; if a single outcome is found in more than $90\%$ cases, communities are accepted and the procedure moves on to the next step; otherwise, the iteration stops, and the partition found at the previous level is returned as the final result. This method is implemented for different configurations of both community detection algorithms, through an in-depth exploration of their parameter space. Moreover, the initial condition required to launch the Leiden algorithm is changed in the different runs, randomly assigning nodes to a random number of communities. The detailed results of such variations are reported and discussed in Supplementary Sec. S5.

### Resolution ratio

In "Rethinking rankings in the framework of development communities" section, we introduced the resolution ratio *R* as a tool to distinguish cases in which the separation between ranked index distributions related to different communities is significant or not. Here, we define this quantity in terms of basic statistical parameters (mean and variance) of the community distributions and the overall distribution. The definition of the resolution ratio *R* is based on the relation between the values $\{x_i\}$ assigned to each element $i=1,\dots ,N$ of a given set and the partition of that set in *K* disjoint groups of cardinality $n_c$, with $c=1,\dots ,K$. Given the values and the partition, one can evaluate the overall mean $\mu$ and variance *D* of the whole set $\{x_i\}$, as well as the mean $\mu _c$ and variance $D_c$ for each group *c*. The key observation^71^ is that the overall variance *D* can be separated in two positive contributions

1 $$\begin{aligned} D=D_{\mathrm {int}} + D_{\mathrm {ext}}, \quad \text {with } D_{\mathrm {int}} = \sum _{c=1}^K \frac{n_c}{N} D_c, \quad D_{\mathrm {ext}} = \sum _{c=1}^K \frac{n_c}{N} (\mu _c-\mu )^2 . \end{aligned}$$

Since $D_{\mathrm {int}}$ coincides with a weighted average of group variances, while $D_{\mathrm {ext}}$ represents the contribution determined by the discrepancy between the group means and the overall mean, a good indicator of separation of group distributions is given by $R=D_{\mathrm {ext}}/D_{\mathrm {int}}$.

### Disclaimer

The designations employed and the presentation of the material in this paper do not imply the expression of any opinion whatsoever on the part of the United Nations concerning the legal status of any country, territory, city or area, or of its authorities, or concerning the delimitation of its frontiers or boundaries. The designations “developed” and “developing” economics are intended for statistical convenience and do not necessarily imply a judgment about the state reached by a particular country or area in the development process. The term “country” as used in the text of this publication also refers, as appropriate, to territories or areas. The views expressed are those of the individual authors of the paper and do not imply any expression of opinion on the part of the United Nations.

## Supplementary information

Supplementary Information.

## Data Availability

The data that support the findings of this study are either publicly available on databases cited in the bibliography, or available from the corresponding author upon reasonable request. The app ERA - Equity-oriented Ranking Analyzer is available upon authentication at https://dashboard.recas.ba.infn.it/ to reproduce the results of this study and customize both input network and rankings.
