# Supplementary material for: An equity-oriented rethink of global rankings with complex networks mapping development
Source: Sci Rep. 2020 Oct 22;10:18046. doi: 10.1038/s41598-020-74964-3 (PMC7582917; doi:10.1038/s41598-020-74964-3)
Supplement: Supplementary file 1 — Supplementary Information. [file 41598_2020_74964_MOESM1_ESM.pdf]

Supplementary Information
An equity-oriented rethink of global rankings with complex networks mapping development

Loredana Bellantuono[1], Alfonso Monaco[2], Sabina Tangaro[3,2], Nicola Amoroso[4,2,*], Vincenzo Aquaro[5], and Roberto Bellotti[1,2]

[1]Dipartimento Interateneo di Fisica "M. Merlin", Università degli studi di Bari "A. Moro", I-70126 Bari, Italy
[2]Istituto Nazionale di Fisica Nucleare, Sezione di Bari, I-70126 Bari, Italy
[3]Dipartimento di Scienze del Suolo, della Pianta e degli Alimenti, Università degli Studi di Bari "A. Moro", Bari, Italy
[4]Dipartimento di Farmacia-Scienze del Farmaco, Università degli studi di Bari "A. Moro", I-70125 Bari, Italy
[5]United Nations Department of Economic and Social Affairs (DESA), Division for Public Institutions and Digital Government, NY 10017 New York, United States
*nicola.amoroso@uniba.it

S1 Country codes

Table S1 reports the ISO 3166-1 alpha-3 codes of UN countries[1], employed throughout the paper.

Table S1. Index of country abbreviations, based on the ISO 3166-1 alpha-3 standard.

Code	Country	Code	Country	Code	Country
AFG	Afghanistan	GMB	Gambia, The	NPL	Nepal
AGO	Angola	GNB	Guinea-Bissau	NRU	Nauru
ALB	Albania	GNQ	Equatorial Guinea	NZL	New Zealand
AND	Andorra	GRC	Greece	OMN	Oman
ARE	United Arab Emirates	GRD	Grenada	PAK	Pakistan
ARG	Argentina	GTM	Guatemala	PAN	Panama
ARM	Armenia	GUY	Guyana	PER	Peru
ATG	Antigua and Barbuda	HND	Honduras	PHL	Philippines
AUS	Australia	HRV	Croatia	PLW	Palau
AUT	Austria	HTI	Haiti	PNG	Papua New Guinea
AZE	Azerbaijan	HUN	Hungary	POL	Poland
BDI	Burundi	IDN	Indonesia	PRK	Korea, Dem. Peoples Rep.
BEL	Belgium	IND	India	PRT	Portugal
BEN	Benin	IRL	Ireland	PRY	Paraguay
BFA	Burkina Faso	IRN	Iran, Islamic Rep.	QAT	Qatar
BGD	Bangladesh	IRQ	Iraq	ROU	Romania
BGR	Bulgaria	ISL	Iceland	RUS	Russian Federation
BHR	Bahrain	ISR	Israel	RWA	Rwanda
BHS	Bahamas, The	ITA	Italy	SAU	Saudi Arabia
BIH	Bosnia and Herzegovina	JAM	Jamaica	SDN	Sudan
BLR	Belarus	JOR	Jordan	SEN	Senegal
BLZ	Belize	JPN	Japan	SGP	Singapore
BOL	Bolivia	KAZ	Kazakhstan	SLB	Solomon Islands
BRA	Brazil	KEN	Kenya	SLE	Sierra Leone
BRB	Barbados	KGZ	Kyrgyz Republic	SLV	El Salvador
BRN	Brunei Darussalam	KHM	Cambodia	SMR	San Marino
BTN	Bhutan	KIR	Kiribati	SOM	Somalia
BWA	Botswana	KNA	St. Kitts and Nevis	SRB	Serbia
CAF	Central African Republic	KOR	Korea, Rep.	SSD	South Sudan
CAN	Canada	KWT	Kuwait	STP	Sao Tome and Principe
CHE	Switzerland	LAO	Lao PDR	SUR	Suriname

CHL	Chile	LBN	Lebanon	SVK	Slovak Republic
CHN	China	LBR	Liberia	SVN	Slovenia
CIV	Cote d'Ivoire	LBY	Libya	SWE	Sweden
CMR	Cameroon	LCA	St. Lucia	SWZ	Eswatini
COD	Congo, Dem. Rep.	LIE	Liechtenstein	SYC	Seychelles
COG	Congo, Rep.	LKA	Sri Lanka	SYR	Syrian Arab Republic
COL	Colombia	LSO	Lesotho	TCD	Chad
COM	Comoros	LTU	Lithuania	TGO	Togo
CPV	Cabo Verde	LUX	Luxembourg	THA	Thailand
CRI	Costa Rica	LVA	Latvia	TJK	Tajikistan
CUB	Cuba	MAR	Morocco	TKM	Turkmenistan
CYP	Cyprus	MCO	Monaco	TLS	Timor-Leste
CZE	Czech Republic	MDA	Moldova	TON	Tonga
DEU	Germany	MDG	Madagascar	TTO	Trinidad and Tobago
DJI	Djibouti	MDV	Maldives	TUN	Tunisia
DMA	Dominica	MEX	Mexico	TUR	Turkey
DNK	Denmark	MHL	Marshall Islands	TUV	Tuvalu
DOM	Dominican Republic	MKD	North Macedonia	TZA	Tanzania
DZA	Algeria	MLI	Mali	UGA	Uganda
ECU	Ecuador	MLT	Malta	UKR	Ukraine
EGY	Egypt, Arab Rep.	MMR	Myanmar	URY	Uruguay
ERI	Eritrea	MNE	Montenegro	USA	United States
ESP	Spain	MNG	Mongolia	UZB	Uzbekistan
EST	Estonia	MOZ	Mozambique	VCT	St. Vincent and the Grenadines
ETH	Ethiopia	MRT	Mauritania	VEN	Venezuela, RB
FIN	Finland	MUS	Mauritius	VNM	Vietnam
FJI	Fiji	MWI	Malawi	VUT	Vanuatu
FRA	France	MYS	Malaysia	WSM	Samoa
FSM	Micronesia, Fed. Sts.	NAM	Namibia	YEM	Yemen, Rep.
GAB	Gabon	NER	Niger	ZAF	South Africa
GBR	United Kingdom	NGA	Nigeria	ZMB	Zambia
GEO	Georgia	NIC	Nicaragua	ZWE	Zimbabwe
GHA	Ghana	NLD	Netherlands		
GIN	Guinea	NOR	Norway		

S2 Selected World Development Indicators

To perform our analysis, we operated a selection of indicators, following criteria of data availability, consistency and non-redundant information. Starting from indicators included in 2016 World Development Indicator (WDI) dataset[2,3] (the most complete involved in the model), we carried out a three-step selection process:

1. from the distribution of available values per indicator, we extracted the subset of 426 indicators with available value for at least 171 states out of 193, corresponding to the 70-percentile of the distribution;

2. we excluded 20 quantities expressed in Local Currency Units, which cannot be directly compared for different states;

3. we computed Pearson correlation between the available values for each pair of residual indicators; if correlation exceeded 0.98, we retained only the indicator with more available values; the third step reduced the number of indicators from 406 to 324, which we called the *selected indicators* or selected WDIs.

The following tables report the selected WDIs, that provide the basis for the network construction, divided by category specified in the UN databases. Countries with missing data for the 2018 network are reported as well. Notice that, due to the low number of indicators, the Financial Sector category is joined with Economic Policy & Debt, the Infrastructure category is joined with Private Sector & Trade, and finally the Education and the Gender categories are joined with Social Protection & Labor.

Environment

Table S2. List of selected WDIs in the Environment category, and respective countries with missing data in the 2018 dataset (with integration from 2017 and 2016).

	Selected WDI	Countries with missing data (2018)
1	Access to clean fuels and technologies for cooking (% of population)	LBN, LBY, LIE, TUR
2	Access to electricity (% of population)	
3	Access to electricity, rural (% of rural population)	NRU
4	Access to electricity, urban (% of urban population)	
5	Agricultural land (% of land area)	MCO, SSD, SDN
6	Agricultural land (sq. km)	MCO
7	Arable land (% of land area)	MCO, NRU, SSD, SDN, TUV
8	Arable land (hectares per person)	ERI, MCO, NRU, SSD, SDN, TUV
9	Arable land (hectares)	MCO, NRU, SSD, TUV
10	Capture fisheries production (metric tons)	
11	Cereal production (metric tons)	AND, BHR, GNQ, KIR, LIE, MHL, MCO, NRU, PLW, WSM, SMR, SYC, SGP, KNA, LCA, TON, TUV
12	Cereal yield (kg per hectare)	AND, BHR, GNQ, ISL, KIR, LIE, MHL, MCO, NRU, PLW, WSM, SMR, SYC, SGP, KNA, LCA, TON, TUV
13	Coal rents (% of GDP)	AND, ERI, PRK, LAO, LIE, MHL, FSM, MCO, NRU, PLW, SMR, SYR, TUV, VEN
14	Crop production index (2004-2006 = 100)	AND, MCO, PLW, SMR, SSD, SDN
15	Food production index (2004-2006 = 100)	AND, MCO, PLW, SMR, SSD, SDN
16	Forest area (% of land area)	MCO, SSD, SDN
17	Forest area (sq. km)	MCO
18	Forest rents (% of GDP)	ERI, PRK, SYR, VEN
19	GDP (constant 2010 US$)	DJI, ERI, PRK, LIE, SOM, SSD, SYR
20	GDP (current US$)	ERI, PRK, SSD, SYR, VEN
21	GDP deflator (base year varies by country)	ERI, PRK, LIE, SOM, SSD, SYR
22	GDP growth (annual %)	ERI, PRK, LIE, SOM, SSD, SYR
23	GDP per capita (constant 2010 US$)	DJI, ERI, PRK, LIE, SOM, SSD, SYR
24	GDP per capita (current US$)	ERI, PRK, SSD, SYR, VEN
25	GDP per capita growth (annual %)	ERI, PRK, LIE, SOM, SSD, SYR
26	GDP per capita, PPP (constant 2011 international $)	AND, CUB, DJI, ERI, PRK, LIE, MCO, SOM, SSD, SYR
27	GDP per capita, PPP (current international $)	AND, CUB, DJI, ERI, PRK, LIE, MCO, SOM, SSD, SYR
28	Land under cereal production (hectares)	AND, BHR, GNQ, ISL, KIR, LIE, MHL, MCO, NRU, PLW, WSM, SMR, SYC, SGP, KNA, LCA, TON, TUV
29	Livestock production index (2004-2006 = 100)	AND, MHL, MCO, PLW, SMR, SSD, SDN
30	Mineral rents (% of GDP)	ERI, PRK, SYR, VEN
31	Natural gas rents (% of GDP)	AND, ERI, PRK, LIE, MHL, FSM, MCO, PLW, SMR, SYR, TUV, VEN
32	Oil rents (% of GDP)	AND, ERI, PRK, LIE, MHL, FSM, MCO, PLW, SMR, SYR, TUV, VEN
33	PM2.5 air pollution, mean annual exposure (micrograms per cubic meter)	LIE, MCO, NRU, PLW, SMR, KNA, TUV
34	PM2.5 air pollution, population exposed to levels exceeding WHO guideline value (% of total)	LIE, MCO, NRU, PLW, SMR, KNA, TUV
35	PM2.5 pollution, population exposed to levels exceeding WHO Interim Target-1 value (% of total)	LIE, MCO, NRU, PLW, SMR, KNA, TUV
36	PM2.5 pollution, population exposed to levels exceeding WHO Interim Target-2 value (% of total)	LIE, MCO, NRU, PLW, SMR, KNA, TUV
37	PM2.5 pollution, population exposed to levels exceeding WHO Interim Target-3 value (% of total)	LIE, MCO, NRU, PLW, SMR, KNA, TUV
38	Permanent cropland (% of land area)	AND, DJI, ISL, LIE, MCO, SMR, SSD, SDN
39	Population density (people per sq. km of land area)	ERI, SSD, SDN
40	Rural population	ERI
41	Rural population (% of total population)	ERI

42	Rural population growth (annual %)	ERI, KWT, MCO, NRU, SGP
43	Surface area (sq. km)	SSD, SDN
44	Terrestrial and marine protected areas (% of total territorial area)	NRU, SMR
45	Terrestrial protected areas (% of total land area)	NRU, SMR
46	Total fisheries production (metric tons)	
47	Total natural resources rents (% of GDP)	ERI, PRK, SYR, VEN
48	Urban population	ERI
49	Urban population (% of total population)	ERI
50	Urban population growth (annual %)	ERI

Economic Policy & Debt, Financial Sector

Table S3. List of selected WDIs in the Economic Policy & Debt and Financial Sectors categories, and respective countries with missing data in the 2018 dataset (with integration from 2017 and 2016).

	Selected WDI	Countries with missing data (2018)
1	Adjusted savings: carbon dioxide damage (% of GNI)	AND, CUB, ERI, PRK, LIE, LTU, MCO, SMR, SRB, SSD, SYR, VEN
2	Adjusted savings: carbon dioxide damage (current US$)	LIE, MCO, SMR, SRB, SSD
3	Adjusted savings: consumption of fixed capital (% of GNI)	AND, CUB, ERI, PRK, LTU, MCO, SMR, SOM, SSD, SYR, VEN
4	Adjusted savings: consumption of fixed capital (current US$)	ERI, PRK, SOM, SSD, SYR, VEN
5	Adjusted savings: education expenditure (% of GNI)	BIH, PRK, MNE, NRU, PLW, SSD, TUV, ARE
6	Adjusted savings: education expenditure (current US$)	AND, BIH, CUB, ERI, PRK, LTU, MCO, MNE, NRU, PLW, SMR, SSD, SYR, TUV, ARE, VEN
7	Adjusted savings: energy depletion (% of GNI)	AND, CUB, ERI, PRK, LIE, LTU, MHL, FSM, MCO, PLW, SMR, SSD, SYR, TUV, VEN
8	Adjusted savings: energy depletion (current US$)	AND, LIE, MHL, FSM, MCO, PLW, SMR, SSD, TUV
9	Adjusted savings: mineral depletion (% of GNI)	AND, CUB, ERI, PRK, LTU, MCO, SMR, SSD, SYR, VEN
10	Adjusted savings: mineral depletion (current US$)	SSD
11	Adjusted savings: natural resources depletion (% of GNI)	AND, ATG, CUB, ERI, GRD, PRK, LIE, LTU, MLT, MHL, FSM, MCO, NRU, PLW, SMR, SSD, KNA, SYR, TKM, TUV, VEN
12	Adjusted savings: net forest depletion (% of GNI)	AND, ATG, CUB, ERI, GRD, PRK, LTU, MLT, MHL, MCO, NRU, PLW, SMR, SSD, KNA, SYR, TKM, TUV, VEN
13	Adjusted savings: net forest depletion (current US$)	AND, ATG, GRD, MLT, MHL, MCO, NRU, PLW, SMR, SSD, KNA, TKM, TUV
14	Adjusted savings: particulate emission damage (current US$)	AND, ATG, DMA, ERI, GRD, KIR, PRK, LIE, MHL, FSM, MCO, NRU, PLW, SMR, SYC, SOM, SSD, KNA, SYR, TUV, VEN
15	Agriculture, forestry, and fishing, value added (% of GDP)	BRB, CAN, ERI, PRK, LBY, LIE, MCO, NRU, SMR, SLB, SOM, SSD, SYR, TKM, TUV, VUT, VEN
16	Agriculture, forestry, and fishing, value added (annual % growth)	AND, ERI, PRK, LBY, LIE, MLT, MCO, NRU, SMR, SLB, SOM, SSD, SYR, TKM, TUV, VUT, VEN
17	Communications, computer, etc. (% of service exports, BoP)	AND, BRB, CAF, TCD, CUB, GNQ, ERI, GAB, IRN, PRK, LIE, FSM, MCO, SMR, SOM, SYR, TKM, TUV, ARE, VNM
18	Communications, computer, etc. (% of service imports, BoP)	AND, BRB, CAF, TCD, CUB, GNQ, ERI, GAB, IRN, PRK, LIE, FSM, MCO, SMR, SOM, SYR, TKM, TUV, ARE, VNM
19	Current account balance (% of GDP)	AND, BRB, CAF, TCD, CUB, GNQ, ERI, GAB, IRN, PRK, LIE, FSM, MCO, SMR, SOM, SSD, SYR, TKM, TUV, ARE, VEN
20	Current account balance (BoP, current US$)	AND, BRB, CAF, TCD, CUB, GNQ, ERI, GAB, IRN, PRK, LIE, FSM, MCO, SMR, SOM, SYR, TKM, TUV, ARE
21	Exports of goods and services (% of GDP)	AND, ERI, FJI, PRK, LIE, MCO, PNG, SMR, STP, SLB, SSD, SUR, SYR, TTO, TUV, VUT, YEM

22	External balance on goods and services (% of GDP)	AND, ERI, FJI, PRK, LIE, MCO, PNG, SMR, STP, SLB, SSD, SUR, SYR, TTO, TUV, VUT, YEM
23	External balance on goods and services (current US$)	AND, ERI, FJI, PRK, LIE, MCO, PNG, SMR, STP, SLB, SSD, SUR, SYR, TTO, TUV, VUT, VEN, YEM
24	Foreign direct investment, net (BoP, current US$)	AND, BRB, CAF, TCD, CUB, GNQ, ERI, GAB, IRN, PRK, LIE, FSM, MCO, SMR, SOM, SYR, TKM, TUV, ARE, YEM
25	Foreign direct investment, net inflows (% of GDP)	AND, CUB, ERI, PRK, FSM, MCO, SMR, SSD, SYR, VEN
26	Foreign direct investment, net inflows (BoP, current US$)	AND, CUB, FSM, MCO, SMR, SYR
27	Foreign direct investment, net outflows (% of GDP)	AND, CUB, ERI, PRK, LIE, FSM, MCO, SMR, SSD, SDN, SYR, VEN
28	Foreign direct investment, net outflows (BoP, current US$)	AND, CUB, LIE, FSM, MCO, SMR, SDN, SYR
29	GDP, PPP (constant 2011 international $)	AND, CUB, DJI, ERI, PRK, LIE, MCO, SOM, SSD, SYR
30	GDP, PPP (current international $)	AND, CUB, DJI, ERI, PRK, LIE, MCO, SOM, SSD, SYR
31	GNI (current US$)	AND, ERI, PRK, MCO, SMR, SSD, SYR, VEN, YEM
32	Imports of goods and services (% of GDP)	AND, ERI, FJI, PRK, LIE, MCO, PNG, SMR, STP, SLB, SSD, SUR, SYR, TTO, TUV, VUT, YEM
33	Industry (including construction), value added (% of GDP)	BRB, CAN, ERI, PRK, LBY, NRU, SMR, SLB, SOM, SSD, SYR, TKM, TUV, VUT, VEN
34	Industry (including construction), value added (annual % growth)	AND, ERI, PRK, LBY, LIE, MLT, MCO, NRU, SMR, SLB, SOM, SSD, SYR, TKM, TUV, VUT
35	Industry (including construction), value added (current US$)	BRB, CAN, ERI, PRK, LBY, NRU, SMR, SLB, SOM, SSD, SYR, TKM, TUV, VUT, VEN
36	Inflation, GDP deflator: linked series (annual %)	ERI, PRK, LIE, SOM, SSD, SYR
37	Insurance and financial services (% of service imports, BoP)	AND, BRB, CAF, TCD, CUB, GNQ, ERI, GAB, IRN, PRK, LIE, FSM, MCO, SMR, SOM, SYR, TKM, TUV, ARE, VNM
38	Net errors and omissions (BoP, current US$)	AND, BRB, CAF, TCD, CUB, GNQ, ERI, GAB, IRN, PRK, LIE, FSM, MCO, SMR, SOM, SYR, TKM, TUV, ARE
39	Net financial account (BoP, current US$)	AND, BRB, CAF, TCD, CUB, GNQ, ERI, GAB, IRN, PRK, LIE, FSM, MCO, SMR, SOM, SYR, TKM, TUV, ARE
40	Net primary income (BoP, current US$)	AND, BRB, CAF, TCD, CUB, GNQ, ERI, GAB, IRN, PRK, LIE, FSM, MCO, SMR, SOM, SYR, TKM, TUV, ARE
41	Net primary income (Net income from abroad) (current US$)	AND, ERI, ISL, PRK, MCO, SMR, SLB, SSD, SYR, VEN
42	Net secondary income (BoP, current US$)	AND, BRB, CAF, TCD, CUB, GNQ, ERI, GAB, IRN, PRK, LIE, FSM, MCO, SMR, SOM, SYR, TKM, TUV, ARE
43	Net secondary income (Net current transfers from abroad) (current US$)	AND, BRN, GNQ, ERI, ISL, IRN, PRK, LIE, MLT, MCO, SMR, SLB, SOM, SSD, SYR, TUV, VUT, VEN
44	Net trade in goods (BoP, current US$)	AND, BRB, CAF, TCD, CUB, GNQ, ERI, GAB, IRN, PRK, LIE, FSM, MCO, SMR, SOM, SYR, TKM, TUV, ARE
45	Personal remittances, received (% of GDP)	AND, BHS, BHR, BRN, CAF, TCD, CUB, GNQ, ERI, PRK, LBY, LIE, MCO, SMR, SGP, SOM, SSD, SYR, ARE, VEN
46	Personal remittances, received (current US$)	AND, BHS, BHR, BRN, CAF, TCD, CUB, GNQ, ERI, PRK, LBY, LIE, MCO, SMR, SGP, SOM, ARE
47	Price level ratio of PPP conversion factor (GDP) to market exchange rate	AND, CUB, DJI, ERI, PRK, LIE, MCO, MMR, SOM, SSD, SYR, VEN
48	Primary income payments (BoP, current US$)	AND, BRB, CAF, TCD, CUB, GNQ, ERI, GAB, IRN, PRK, LIE, FSM, MCO, SMR, SOM, SYR, TKM, TUV, ARE
49	Primary income receipts (BoP, current US$)	AND, BRB, CAF, TCD, CUB, GNQ, ERI, GAB, IRN, PRK, LIE, FSM, MCO, SMR, SOM, SYR, TKM, TUV, ARE
50	Reserves and related items (BoP, current US$)	AND, BRB, CAF, TCD, CUB, GNQ, ERI, GAB, IRN, PRK, LIE, FSM, MCO, SMR, SOM, SYR, TKM, TUV, ARE
51	Service exports (BoP, current US$)	AND, BRB, CAF, TCD, CUB, GNQ, ERI, GAB, IRN, PRK, LIE, FSM, MCO, SMR, SOM, SYR, TKM, TUV, ARE
52	Service imports (BoP, current US$)	AND, BRB, CAF, TCD, CUB, GNQ, ERI, GAB, IRN, PRK, LIE, FSM, MCO, SMR, SOM, SYR, TKM, TUV, ARE

53	Services, value added (% of GDP)	BRB, CAN, ERI, PRK, LBY, LIE, NRU, SMR, SLB, SOM, SSD, SYR, TON, TKM, TUV, VUT, VEN
54	Services, value added (annual % growth)	AND, ERI, PRK, LBY, LIE, MLT, MCO, NRU, SMR, SLB, SOM, SSD, SYR, TON, TKM, TUV, VUT, VEN
55	Trade (% of GDP)	AND, ERI, FJI, PRK, LIE, MCO, PNG, SMR, STP, SLB, SSD, SUR, SYR, TTO, TUV, VUT, YEM
56	Trade in services (% of GDP)	AND, BRB, CAF, TCD, CUB, GNQ, ERI, GAB, IRN, PRK, LIE, FSM, MCO, SMR, SOM, SSD, SYR, TKM, TUV, ARE, VEN
57	Travel services (% of service imports, BoP)	AND, BRB, CAF, TCD, CUB, GNQ, ERI, GAB, IRN, PRK, LIE, FSM, MCO, SMR, SOM, SYR, TKM, TUV, ARE, VNM

Health

Table S4. List of selected WDIs in the Health category, and respective countries with missing data in the 2018 dataset (with integration from 2017 and 2016).

	Selected WDI	Countries with missing data (2018)
1	Adolescent fertility rate (births per 1,000 women ages 15-19)	AND, DMA, LIE, MHL, MCO, NRU, PLW, SMR, KNA, TUV
2	Age dependency ratio (% of working-age population)	AND, DMA, ERI, LIE, MHL, MCO, NRU, PLW, SMR, KNA, TUV
3	Birth rate, crude (per 1,000 people)	DMA, MHL, NRU, KNA, TUV
4	Cause of death, by communicable diseases and maternal, prenatal and nutrition conditions (% of total)	AND, DMA, LIE, MHL, MCO, NRU, PLW, SMR, KNA, TUV
5	Cause of death, by injury (% of total)	AND, DMA, LIE, MHL, MCO, NRU, PLW, SMR, KNA, TUV
6	Cause of death, by non-communicable diseases (% of total)	AND, DMA, LIE, MHL, MCO, NRU, PLW, SMR, KNA, TUV
7	Current health expenditure (% of GDP)	PRK, LBY, LIE, SOM, SSD, SYR, YEM
8	Death rate, crude (per 1,000 people)	DMA, MHL, NRU, KNA, TUV
9	Domestic general government health expenditure (% of GDP)	PRK, LBY, LIE, SOM, SSD, SYR, YEM
10	Domestic general government health expenditure (% of current health expenditure)	PRK, LBY, LIE, SOM, SSD, SYR, YEM
11	Domestic general government health expenditure (% of general government expenditure)	PRK, LIE, SOM, SSD, SYR
12	Domestic general government health expenditure per capita (current US$)	PRK, LBY, LIE, SOM, SSD, SYR, VEN, YEM
13	Domestic general government health expenditure per capita, PPP (current international $)	IRQ, PRK, LBY, LIE, SOM, SSD, SYR, VEN, YEM
14	Domestic private health expenditure (% of current health expenditure)	PRK, LBY, LIE, SOM, SSD, SYR, YEM
15	Domestic private health expenditure per capita (current US$)	PRK, LBY, LIE, SOM, SSD, SYR, VEN, YEM
16	Domestic private health expenditure per capita, PPP (current international $)	IRQ, PRK, LBY, LIE, SOM, SSD, SYR, VEN, YEM
17	Fertility rate, total (births per woman)	AND, DMA, MHL, MCO, NRU, PLW, SMR, KNA, TUV
18	Immunization, DPT (% of children ages 12-23 months)	LIE
19	Immunization, HepB3 (% of one-year-old children)	DNK, FIN, HUN, ISL, JPN, LIE, NOR, SVN, GBR
20	Immunization, measles (% of children ages 12-23 months)	LIE
21	Incidence of tuberculosis (per 100,000 people)	LIE
22	Life expectancy at birth, female (years)	AND, DMA, MHL, MCO, NRU, PLW, SMR, KNA, TUV
23	Life expectancy at birth, male (years)	AND, DMA, MHL, MCO, NRU, PLW, SMR, KNA, TUV
24	Life expectancy at birth, total (years)	AND, DMA, MHL, MCO, NRU, PLW, SMR, KNA, TUV
25	Lifetime risk of maternal death (%)	AND, DMA, LIE, MHL, MCO, NRU, PLW, SMR, KNA, TUV
26	Lifetime risk of maternal death (1 in: rate varies by country)	AND, DMA, LIE, MHL, MCO, NRU, PLW, SMR, KNA, TUV

27	Maternal mortality ratio (modeled estimate, per 100,000 live births)	AND, DMA, LIE, MHL, MCO, NRU, PLW, SMR, KNA, TUV
28	Mortality caused by road traffic injury (per 100,000 people)	DZA, AND, BHS, BHR, BRN, DJI, HTI, PRK, LIE, MHL, MCO, NRU, NIC, PLW, SLE, KNA, VCT, TUV, YEM, ZMB
29	Mortality from CVD, cancer, diabetes or CRD between exact ages 30 and 70 (%)	AND, DMA, LIE, MHL, MCO, NRU, PLW, SMR, KNA, TUV
30	Mortality from CVD, cancer, diabetes or CRD between exact ages 30 and 70, female (%)	AND, DMA, LIE, MHL, MCO, NRU, PLW, SMR, KNA, TUV
31	Mortality from CVD, cancer, diabetes or CRD between exact ages 30 and 70, male (%)	AND, DMA, LIE, MHL, MCO, NRU, PLW, SMR, KNA, TUV
32	Mortality rate attributed to household and ambient air pollution, age-standardized, female (per 100,000 female population)	AND, DMA, LIE, MHL, MCO, NRU, PLW, SMR, KNA, TUV
33	Mortality rate attributed to household and ambient air pollution, age-standardized, male (per 100,000 male population)	AND, DMA, LIE, MHL, MCO, NRU, PLW, SMR, KNA, TUV
34	Mortality rate attributed to unintentional poisoning, female (per 100,000 female population)	AND, DMA, LIE, MHL, MCO, NRU, PLW, SMR, KNA, TUV
35	Mortality rate attributed to unintentional poisoning, male (per 100,000 male population)	AND, DMA, LIE, MHL, MCO, NRU, PLW, SMR, KNA, TUV
36	Mortality rate attributed to unsafe water, unsafe sanitation and lack of hygiene (per 100,000 population)	AND, DMA, LIE, MHL, MCO, NRU, PLW, SMR, KNA, TUV
37	Mortality rate, neonatal (per 1,000 live births)	LIE
38	Mortality rate, under-5 (per 1,000 live births)	LIE
39	Number of maternal deaths	AND, DMA, LIE, MHL, MCO, NRU, PLW, SMR, KNA, TUV
40	Number of neonatal deaths	LIE
41	Number of under-five deaths	LIE
42	Out-of-pocket expenditure (% of current health expenditure)	PRK, LBY, LIE, SOM, SSD, SYR, YEM
43	Out-of-pocket expenditure per capita (current US$)	PRK, LBY, LIE, SOM, SSD, SYR, VEN, YEM
44	Out-of-pocket expenditure per capita, PPP (current international $)	IRQ, PRK, LBY, LIE, SOM, SSD, SYR, VEN, YEM
45	People practicing open defecation (% of population)	ARG, BRN, HRV, DMA, FSM, KNA
46	People using at least basic drinking water services (% of population)	DMA, KNA
47	People using at least basic sanitation services (% of population)	BRN, DMA, KNA
48	Population ages 05-09, female (% of female population)	AND, DMA, ERI, LIE, MHL, MCO, NRU, PLW, SMR, KNA, TUV
49	Population ages 05-09, male (% of male population)	AND, DMA, ERI, LIE, MHL, MCO, NRU, PLW, SMR, KNA, TUV
50	Population ages 10-14, female (% of female population)	AND, DMA, ERI, LIE, MHL, MCO, NRU, PLW, SMR, KNA, TUV
51	Population ages 10-14, male (% of male population)	AND, DMA, ERI, LIE, MHL, MCO, NRU, PLW, SMR, KNA, TUV
52	Population ages 15-19, female (% of female population)	AND, DMA, ERI, LIE, MHL, MCO, NRU, PLW, SMR, KNA, TUV
53	Population ages 15-19, male (% of male population)	AND, DMA, ERI, LIE, MHL, MCO, NRU, PLW, SMR, KNA, TUV
54	Population ages 15-64, female (% of female population)	AND, DMA, ERI, LIE, MHL, MCO, NRU, PLW, SMR, KNA, TUV
55	Population ages 15-64, male (% of male population)	AND, DMA, ERI, LIE, MHL, MCO, NRU, PLW, SMR, KNA, TUV
56	Population ages 20-24, female (% of female population)	AND, DMA, ERI, LIE, MHL, MCO, NRU, PLW, SMR, KNA, TUV
57	Population ages 20-24, male (% of male population)	AND, DMA, ERI, LIE, MHL, MCO, NRU, PLW, SMR, KNA, TUV

58	Population ages 25-29, female (% of female population)	AND, DMA, ERI, LIE, MHL, MCO, NRU, PLW, SMR, KNA, TUV
59	Population ages 25-29, male (% of male population)	AND, DMA, ERI, LIE, MHL, MCO, NRU, PLW, SMR, KNA, TUV
60	Population ages 30-34, female (% of female population)	AND, DMA, ERI, LIE, MHL, MCO, NRU, PLW, SMR, KNA, TUV
61	Population ages 30-34, male (% of male population)	AND, DMA, ERI, LIE, MHL, MCO, NRU, PLW, SMR, KNA, TUV
62	Population ages 35-39, female (% of female population)	AND, DMA, ERI, LIE, MHL, MCO, NRU, PLW, SMR, KNA, TUV
63	Population ages 35-39, male (% of male population)	AND, DMA, ERI, LIE, MHL, MCO, NRU, PLW, SMR, KNA, TUV
64	Population ages 40-44, female (% of female population)	AND, DMA, ERI, LIE, MHL, MCO, NRU, PLW, SMR, KNA, TUV
65	Population ages 40-44, male (% of male population)	AND, DMA, ERI, LIE, MHL, MCO, NRU, PLW, SMR, KNA, TUV
66	Population ages 45-49, female (% of female population)	AND, DMA, ERI, LIE, MHL, MCO, NRU, PLW, SMR, KNA, TUV
67	Population ages 45-49, male (% of male population)	AND, DMA, ERI, LIE, MHL, MCO, NRU, PLW, SMR, KNA, TUV
68	Population ages 50-54, female (% of female population)	AND, DMA, ERI, LIE, MHL, MCO, NRU, PLW, SMR, KNA, TUV
69	Population ages 50-54, male (% of male population)	AND, DMA, ERI, LIE, MHL, MCO, NRU, PLW, SMR, KNA, TUV
70	Population ages 55-59, male (% of male population)	AND, DMA, ERI, LIE, MHL, MCO, NRU, PLW, SMR, KNA, TUV
71	Population ages 60-64, male (% of male population)	AND, DMA, ERI, LIE, MHL, MCO, NRU, PLW, SMR, KNA, TUV
72	Population ages 65-69, male (% of male population)	AND, DMA, ERI, LIE, MHL, MCO, NRU, PLW, SMR, KNA, TUV
73	Population ages 70-74, female (% of female population)	AND, DMA, ERI, LIE, MHL, MCO, NRU, PLW, SMR, KNA, TUV
74	Population ages 70-74, male (% of male population)	AND, DMA, ERI, LIE, MHL, MCO, NRU, PLW, SMR, KNA, TUV
75	Population ages 75-79, female (% of female population)	AND, DMA, ERI, LIE, MHL, MCO, NRU, PLW, SMR, KNA, TUV
76	Population ages 75-79, male (% of male population)	AND, DMA, ERI, LIE, MHL, MCO, NRU, PLW, SMR, KNA, TUV
77	Population ages 80 and above, female (% of female population)	AND, DMA, ERI, LIE, MHL, MCO, NRU, PLW, SMR, KNA, TUV
78	Population ages 80 and above, male (% of male population)	AND, DMA, ERI, LIE, MHL, MCO, NRU, PLW, SMR, KNA, TUV
79	Population growth (annual %)	ERI
80	Population, female (% of total population)	AND, DMA, ERI, LIE, MHL, MCO, NRU, PLW, SMR, KNA, TUV
81	Population, male (% of total population)	AND, DMA, ERI, LIE, MHL, MCO, NRU, PLW, SMR, KNA, TUV
82	Population, total	ERI
83	Prevalence of anemia among children (% of children under 5)	LIE, MCO, NRU, PLW, SMR, KNA, TUV
84	Prevalence of anemia among pregnant women (%)	LIE, MCO, NRU, PLW, SMR, KNA, TUV
85	Prevalence of anemia among women of reproductive age (% of women ages 15-49)	LIE, MCO, NRU, PLW, SMR, KNA, TUV
86	Sex ratio at birth (male births per female births)	AND, DMA, LIE, MHL, MCO, NRU, PLW, SMR, KNA, TUV
87	Suicide mortality rate (per 100,000 population)	AND, DMA, LIE, MHL, MCO, NRU, PLW, SMR, KNA, TUV
88	Suicide mortality rate, female (per 100,000 female population)	AND, DMA, LIE, MHL, MCO, NRU, PLW, SMR, KNA, TUV

89	Suicide mortality rate, male (per 100,000 male population)	AND, DMA, LIE, MHL, MCO, NRU, PLW, SMR, KNA, TUV
90	Total alcohol consumption per capita, female (liters of pure alcohol, projected estimates, female 15+ years of age)	LIE, MHL, MCO, PLW, SMR, SSD
91	Total alcohol consumption per capita, male (liters of pure alcohol, projected estimates, male 15+ years of age)	LIE, MHL, MCO, PLW, SMR, SSD
92	Tuberculosis case detection rate (%, all forms)	LIE, MCO, SMR
93	Tuberculosis treatment success rate (% of new cases)	GRC, ITA, LIE, MLT, MCO, SMR

Private Sector & Trade, Infrastructure

Table S5. List of selected WDIs in the Private Sector & Trade and Infrastructure categories, and respective countries with missing data in the 2018 dataset (with integration from 2017 and 2016).

	Selected WDI	Countries with missing data (2018)
1	Business extent of disclosure index (0=less disclosure to 10=more disclosure)	AND, CUB, PRK, MCO, NRU, TKM, TUV
2	Computer, communications and other services (% of commercial service exports)	AND, BRB, CAF, TCD, CUB, GNQ, ERI, GAB, IRN, PRK, LIE, FSM, MCO, SMR, SOM, SYR, TKM, TUV, ARE, VNM
3	Computer, communications and other services (% of commercial service imports)	AND, BRB, CAF, TCD, CUB, GNQ, ERI, GAB, IRN, PRK, LIE, FSM, MCO, SMR, SOM, SYR, TKM, TUV, ARE, VNM
4	Cost of business start-up procedures (% of GNI per capita)	AND, CUB, PRK, MCO, NRU, TKM, TUV
5	Cost of business start-up procedures, male (% of GNI per capita)	AND, CUB, PRK, MCO, NRU, TKM, TUV
6	Cost to export, border compliance (US$)	AND, CUB, ERI, PRK, MCO, NRU, TKM, TUV, YEM
7	Cost to export, documentary compliance (US$)	AND, CUB, ERI, PRK, MCO, NRU, TKM, TUV, YEM
8	Cost to import, border compliance (US$)	AND, CUB, ERI, PRK, MCO, NRU, TKM, TUV, YEM
9	Cost to import, documentary compliance (US$)	AND, CUB, ERI, PRK, MCO, NRU, TKM, TUV, YEM
10	Depth of credit information index (0=low to 8=high)	AND, CUB, PRK, MCO, NRU, TKM, TUV
11	Ease of doing business score (0 = lowest performance to 100 = best performance)	AND, CUB, PRK, MCO, NRU, TKM, TUV
12	Export unit value index (2000 = 100)	LIE, MCO, MNE, SMR, SSD, TLS, TUV
13	Export value index (2000 = 100)	LIE, MCO, MNE, SMR, SSD, TLS, TUV
14	Export volume index (2000 = 100)	LIE, MCO, MNE, SMR, SSD, TLS, TUV
15	Fixed broadband subscriptions	COG, PRK, NRU, PLW, SLE
16	Fixed broadband subscriptions (per 100 people)	COG, PRK, NRU, PLW, SLE
17	Fixed telephone subscriptions	MHL, NRU, PLW
18	Fixed telephone subscriptions (per 100 people)	MHL, NRU, PLW
19	Import unit value index (2000 = 100)	AND, LIE, MCO, MNE, SMR, SSD, TLS, TUV
20	Import value index (2000 = 100)	LIE, MCO, MNE, SMR, SSD, TLS, TUV
21	Import volume index (2000 = 100)	AND, LIE, MCO, MNE, SMR, SSD, TLS, TUV
22	Individuals using the Internet (% of population)	PRK, PLW
23	International tourism, expenditures (% of total imports)	AND, BRB, CAF, TCD, CUB, GNQ, ERI, GAB, IRN, PRK, LIE, FSM, MCO, SMR, SOM, SYR, TKM, TUV, ARE
24	International tourism, expenditures (current US$)	AND, BRB, CAF, TCD, CUB, GNQ, ERI, PRK, LIE, FSM, MCO, SMR, SOM, SYR, TKM, TUV
25	International tourism, number of arrivals	AFG, DJI, GNQ, GAB, GHA, IRQ, PRK, LBR, LBY, MRT, NRU, PAK, SOM, SSD, SYR, TKM, YEM
26	International tourism, receipts (% of total exports)	AND, BRB, CAF, TCD, CUB, GNQ, ERI, GAB, IRN, PRK, LBR, LBY, LIE, FSM, MCO, SMR, SOM, SYR, TKM, TUV, ARE
27	International tourism, receipts (current US$)	AND, CAF, TCD, GNQ, PRK, LBR, LBY, LIE, FSM, MCO, SMR, SOM, SYR, TKM, TUV
28	Labor tax and contributions (% of commercial profits)	AND, CUB, PRK, MCO, NRU, SOM, TKM, TUV
29	Merchandise exports by the reporting economy (current US$)	AND, LIE, MCO

30	Merchandise exports by the reporting economy, residual (% of total merchandise exports)	AND, LIE, MCO
31	Merchandise exports to economies in the Arab World (% of total merchandise exports)	AND, LIE, MCO
32	Merchandise exports to high-income economies (% of total merchandise exports)	AND, LIE, MCO
33	Merchandise exports to low- and middle-income economies in East Asia & Pacific (% of total merchandise exports)	AND, LIE, MCO
34	Merchandise exports to low- and middle-income economies in Europe & Central Asia (% of total merchandise exports)	AND, KIR, LIE, MCO
35	Merchandise exports to low- and middle-income economies in Latin America & the Caribbean (% of total merchandise exports)	AFG, AND, KIR, LIE, MCO
36	Merchandise exports to low- and middle-income economies in Middle East & North Africa (% of total merchandise exports)	AND, LIE, MDV, MCO, KNA
37	Merchandise exports to low- and middle-income economies in South Asia (% of total merchandise exports)	AND, LIE, MCO
38	Merchandise exports to low- and middle-income economies in Sub-Saharan Africa (% of total merchandise exports)	AND, KIR, LIE, MCO
39	Merchandise exports to low- and middle-income economies outside region (% of total merchandise exports)	AND, LIE, MCO
40	Merchandise imports by the reporting economy (current US$)	AND, LIE, MCO
41	Merchandise imports by the reporting economy, residual (% of total merchandise imports)	AND, LIE, MCO
42	Merchandise imports from economies in the Arab World (% of total merchandise imports)	AND, LIE, MCO
43	Merchandise imports from high-income economies (% of total merchandise imports)	AND, LIE, MCO
44	Merchandise imports from low- and middle-income economies in East Asia & Pacific (% of total merchandise imports)	AND, LIE, MCO
45	Merchandise imports from low- and middle-income economies in Europe & Central Asia (% of total merchandise imports)	AND, LIE, MCO
46	Merchandise imports from low- and middle-income economies in Latin America & the Caribbean (% of total merchandise imports)	AND, LIE, MCO
47	Merchandise imports from low- and middle-income economies in Middle East & North Africa (% of total merchandise imports)	AND, LIE, MCO, TUV
48	Merchandise imports from low- and middle-income economies in South Asia (% of total merchandise imports)	AND, LIE, MCO
49	Merchandise imports from low- and middle-income economies in Sub-Saharan Africa (% of total merchandise imports)	AND, LIE, MCO, SYR
50	Merchandise imports from low- and middle-income economies outside region (% of total merchandise imports)	AND, LIE, MCO
51	Merchandise trade (% of GDP)	AND, ERI, PRK, LIE, MCO, SMR, SOM, SSD, SYR, TUV, VEN

52	Mobile cellular subscriptions (per 100 people)	PLW
53	Net barter terms of trade index (2000 = 100)	AND, LIE, MCO, MNE, SMR, SSD, TLS, TUV
54	Other taxes payable by businesses (% of commercial profits)	AND, CUB, PRK, MCO, NRU, SOM, TKM, TUV
55	Private credit bureau coverage (% of adults)	AND, CUB, PRK, MCO, NRU, TKM, TUV
56	Procedures to build a warehouse (number)	AND, CUB, ERI, PRK, LBY, MCO, NRU, SOM, SYR, TKM, TUV, YEM
57	Procedures to register property (number)	AND, CUB, PRK, LBY, MHL, FSM, MCO, NRU, TLS, TKM, TUV
58	Profit tax (% of commercial profits)	AND, CUB, PRK, MCO, NRU, SOM, TKM, TUV
59	Public credit registry coverage (% of adults)	AND, CUB, PRK, MCO, NRU, TKM, TUV
60	Scientific and technical journal articles	
61	Secure Internet servers	
62	Secure Internet servers (per 1 million people)	ERI
63	Start-up procedures to register a business, male (number)	AND, CUB, PRK, MCO, NRU, TKM, TUV
64	Strength of legal rights index (0=weak to 12=strong)	AND, CUB, PRK, MCO, NRU, TKM, TUV
65	Tax payments (number)	AND, CUB, PRK, MCO, NRU, SOM, TKM, TUV
66	Time required to build a warehouse (days)	AND, CUB, ERI, PRK, LBY, MCO, NRU, SOM, SYR, TKM, TUV, YEM
67	Time required to enforce a contract (days)	AND, CUB, PRK, MCO, NRU, TKM, TUV
68	Time required to get electricity (days)	AND, CUB, ERI, PRK, MCO, NRU, SOM, SSD, TKM, TUV, YEM
69	Time required to register property (days)	AND, CUB, PRK, LBY, MHL, FSM, MCO, NRU, TLS, TKM, TUV
70	Time required to start a business, male (days)	AND, CUB, PRK, MCO, NRU, TKM, TUV
71	Time to export, border compliance (hours)	AND, CUB, ERI, PRK, MCO, NRU, TKM, TUV, YEM
72	Time to export, documentary compliance (hours)	AND, CUB, ERI, PRK, MCO, NRU, TKM, TUV, YEM
73	Time to import, border compliance (hours)	AND, CUB, ERI, PRK, MCO, NRU, TKM, TUV, YEM
74	Time to import, documentary compliance (hours)	AND, CUB, ERI, PRK, MCO, NRU, TKM, TUV, YEM
75	Time to prepare and pay taxes (hours)	AND, CUB, PRK, MCO, NRU, SOM, TKM, TUV
76	Total tax and contribution rate (% of profit)	AND, CUB, PRK, MCO, NRU, SOM, TKM, TUV
77	Transport services (% of service imports, BoP)	AND, BRB, CAF, TCD, CUB, GNQ, ERI, GAB, IRN, PRK, LIE, FSM, MCO, SMR, SOM, SYR, TKM, TUV, ARE, VNM

Social Protection & Labor, Education, Gender

Table S6. List of selected WDIs in the Social Protection & Labor, Education and Gender categories, and respective countries with missing data in the 2018 dataset (with integration from 2017 and 2016).

	Selected WDI	Countries with missing data (2018)
1	Compulsory education, duration (years)	BTN, BWA, BDI, KHM, FJI, MDV, FSM, MOZ, NER, PNG, SLB, SOM, VUT
2	Contributing family workers, female (% of female employment) (modeled ILO estimate)	AND, ATG, DMA, GRD, KIR, LIE, MHL, FSM, MCO, NRU, PLW, SMR, SYC, KNA, TUV
3	Contributing family workers, male (% of male employment) (modeled ILO estimate)	AND, ATG, DMA, GRD, KIR, LIE, MHL, FSM, MCO, NRU, PLW, SMR, SYC, KNA, TUV
4	Contributing family workers, total (% of total employment) (modeled ILO estimate)	AND, ATG, DMA, GRD, KIR, LIE, MHL, FSM, MCO, NRU, PLW, SMR, SYC, KNA, TUV
5	Employers, female (% of female employment) (modeled ILO estimate)	AND, ATG, DMA, GRD, KIR, LIE, MHL, FSM, MCO, NRU, PLW, SMR, SYC, KNA, TUV
6	Employers, total (% of total employment) (modeled ILO estimate)	AND, ATG, DMA, GRD, KIR, LIE, MHL, FSM, MCO, NRU, PLW, SMR, SYC, KNA, TUV
7	Employment in agriculture, female (% of female employment) (modeled ILO estimate)	AND, ATG, DMA, GRD, KIR, LIE, MHL, FSM, MCO, NRU, PLW, SMR, SYC, KNA, TUV
8	Employment in agriculture, male (% of male employment) (modeled ILO estimate)	AND, ATG, DMA, GRD, KIR, LIE, MHL, FSM, MCO, NRU, PLW, SMR, SYC, KNA, TUV
9	Employment in industry (% of total employment) (modeled ILO estimate)	AND, ATG, DMA, GRD, KIR, LIE, MHL, FSM, MCO, NRU, PLW, SMR, SYC, KNA, TUV

10	Employment in industry, female (% of female employment) (modeled ILO estimate)	AND, ATG, DMA, GRD, KIR, LIE, MHL, FSM, MCO, NRU, PLW, SMR, SYC, KNA, TUV
11	Employment in industry, male (% of male employment) (modeled ILO estimate)	AND, ATG, DMA, GRD, KIR, LIE, MHL, FSM, MCO, NRU, PLW, SMR, SYC, KNA, TUV
12	Employment in services (% of total employment) (modeled ILO estimate)	AND, ATG, DMA, GRD, KIR, LIE, MHL, FSM, MCO, NRU, PLW, SMR, SYC, KNA, TUV
13	Employment in services, female (% of female employment) (modeled ILO estimate)	AND, ATG, DMA, GRD, KIR, LIE, MHL, FSM, MCO, NRU, PLW, SMR, SYC, KNA, TUV
14	Employment in services, male (% of male employment) (modeled ILO estimate)	AND, ATG, DMA, GRD, KIR, LIE, MHL, FSM, MCO, NRU, PLW, SMR, SYC, KNA, TUV
15	Employment to population ratio, 15+, male (%) (modeled ILO estimate)	AND, ATG, DMA, GRD, KIR, LIE, MHL, FSM, MCO, NRU, PLW, SMR, SYC, KNA, TUV
16	Employment to population ratio, 15+, total (%) (modeled ILO estimate)	AND, ATG, DMA, GRD, KIR, LIE, MHL, FSM, MCO, NRU, PLW, SMR, SYC, KNA, TUV
17	Employment to population ratio, ages 15-24, female (%) (modeled ILO estimate)	AND, ATG, DMA, GRD, KIR, LIE, MHL, FSM, MCO, NRU, PLW, SMR, SYC, KNA, TUV
18	Employment to population ratio, ages 15-24, male (%) (modeled ILO estimate)	AND, ATG, DMA, GRD, KIR, LIE, MHL, FSM, MCO, NRU, PLW, SMR, SYC, KNA, TUV
19	Employment to population ratio, ages 15-24, total (%) (modeled ILO estimate)	AND, ATG, DMA, GRD, KIR, LIE, MHL, FSM, MCO, NRU, PLW, SMR, SYC, KNA, TUV
20	GDP per person employed (constant 2011 PPP $)	AND, ATG, CUB, DMA, GRD, KIR, LIE, MHL, FSM, MCO, NRU, PLW, SMR, SYC, KNA, TUV
21	Labor force participation rate for ages 15-24, female (%) (modeled ILO estimate)	AND, ATG, DMA, GRD, KIR, LIE, MHL, FSM, MCO, NRU, PLW, SMR, SYC, KNA, TUV
22	Labor force participation rate for ages 15-24, male (%) (modeled ILO estimate)	AND, ATG, DMA, GRD, KIR, LIE, MHL, FSM, MCO, NRU, PLW, SMR, SYC, KNA, TUV
23	Labor force participation rate for ages 15-24, total (%) (modeled ILO estimate)	AND, ATG, DMA, GRD, KIR, LIE, MHL, FSM, MCO, NRU, PLW, SMR, SYC, KNA, TUV
24	Labor force participation rate, female (% of female population ages 15+) (modeled ILO estimate)	AND, ATG, DMA, GRD, KIR, LIE, MHL, FSM, MCO, NRU, PLW, SMR, SYC, KNA, TUV
25	Labor force participation rate, female (% of female population ages 15-64) (modeled ILO estimate)	AND, ATG, DMA, GRD, KIR, LIE, MHL, FSM, MCO, NRU, PLW, SMR, SYC, KNA, TUV
26	Labor force participation rate, male (% of male population ages 15+) (modeled ILO estimate)	AND, ATG, DMA, GRD, KIR, LIE, MHL, FSM, MCO, NRU, PLW, SMR, SYC, KNA, TUV
27	Labor force participation rate, male (% of male population ages 15-64) (modeled ILO estimate)	AND, ATG, DMA, GRD, KIR, LIE, MHL, FSM, MCO, NRU, PLW, SMR, SYC, KNA, TUV
28	Labor force participation rate, total (% of total population ages 15+) (modeled ILO estimate)	AND, ATG, DMA, GRD, KIR, LIE, MHL, FSM, MCO, NRU, PLW, SMR, SYC, KNA, TUV
29	Labor force participation rate, total (% of total population ages 15-64) (modeled ILO estimate)	AND, ATG, DMA, GRD, KIR, LIE, MHL, FSM, MCO, NRU, PLW, SMR, SYC, KNA, TUV
30	Labor force, female (% of total labor force)	AND, ATG, DMA, ERI, GRD, KIR, LIE, MHL, FSM, MCO, NRU, PLW, SMR, SYC, KNA, TUV
31	Law mandates equal remuneration for females and males for work of equal value (1=yes; 0=no)	AND, CUB, PRK, LIE, MCO, NRU, SOM, TKM, TUV, VEN, YEM
32	Legislation exists on domestic violence (1=yes; 0=no)	AND, CUB, PRK, LIE, MCO, NRU, SOM, TKM, TUV, VEN, YEM
33	Lower secondary school starting age (years)	
34	Preprimary education, duration (years)	USA
35	Primary education, duration (years)	
36	Primary school starting age (years)	
37	Proportion of seats held by women in national parliaments (%)	
38	Ratio of female to male labor force participation rate (%) (modeled ILO estimate)	AND, ATG, DMA, GRD, KIR, LIE, MHL, FSM, MCO, NRU, PLW, SMR, SYC, KNA, TUV
39	Refugee population by country or territory of origin	LIE, FSM, NRU
40	Secondary education, duration (years)	
41	Unemployment, female (% of female labor force) (modeled ILO estimate)	AND, ATG, DMA, GRD, KIR, LIE, MHL, FSM, MCO, NRU, PLW, SMR, SYC, KNA, TUV

42	Unemployment, male (% of male labor force) (modeled ILO estimate)	AND, ATG, DMA, GRD, KIR, LIE, MHL, FSM, MCO, NRU, PLW, SMR, SYC, KNA, TUV
43	Unemployment, total (% of total labor force) (modeled ILO estimate)	AND, ATG, DMA, GRD, KIR, LIE, MHL, FSM, MCO, NRU, PLW, SMR, SYC, KNA, TUV
44	Unemployment, youth female (% of female labor force ages 15-24) (modeled ILO estimate)	AND, ATG, DMA, GRD, KIR, LIE, MHL, FSM, MCO, NRU, PLW, SMR, SYC, KNA, TUV
45	Unemployment, youth total (% of total labor force ages 15-24) (modeled ILO estimate)	AND, ATG, DMA, GRD, KIR, LIE, MHL, FSM, MCO, NRU, PLW, SMR, SYC, KNA, TUV
46	Vulnerable employment, total (% of total employment) (modeled ILO estimate)	AND, ATG, DMA, GRD, KIR, LIE, MHL, FSM, MCO, NRU, PLW, SMR, SYC, KNA, TUV
47	Wage and salaried workers, total (% of total employment) (modeled ILO estimate)	AND, ATG, DMA, GRD, KIR, LIE, MHL, FSM, MCO, NRU, PLW, SMR, SYC, KNA, TUV

S3 Data availability

We report here a synthesis of the results on WDI availability for the 2018, 2015, 2012 and 2009 data set. In particular, Tab. S7 shows all the cases in which the fraction of missing data in a certain data set (integrated by data from the two years before the nominal one) is larger than 30%. Moreover, we show in bar plots the fractions of missing data, data obtained for the nominal year, and data retrieved from the two years before, for the 2018 (Fig. S1), 2015 (Fig. S2) and 2012 (Fig. S3) data sets. We omit the diagram representing data availability for 2009 as a nominal year, since in this case we recognized that the available information was insufficient to perform a robust analysis.

Table S7. The table reports cases in which the percentage of missing data for a country in a data set is larger than 30%. The values are always referred to data from the nominal year, integrated with information from the two previous years.

**Country**	**Missing data per year (%)**			
	**2018**	**2015**	**2012**	**2009**
Andorra	68.21	69.44	68.52	70.99
Dominica	31.17	30.86	33.93	37.35
Eritrea	39.81	41.98		
Dem. People's Rep. Korea	37.96	39.81	39.19	45.37
Liechtenstein	68.52	77.78	77.78	77.78
Marshall Islands	36.11	36.11	40.43	43.52
Monaco	76.23	75.62	76.54	79.01
Nauru	51.85	50.62	50.62	54.94
Palau	39.81	38.27	42.59	42.90
San Marino	56.79	57.10	59.88	66.05
Somalia			38.27	45.06
South Sudan				56.79
St. Kitts and Nevis	36.11	35.80	38.89	40.12
Tuvalu	62.35	54.94	54.01	54.94

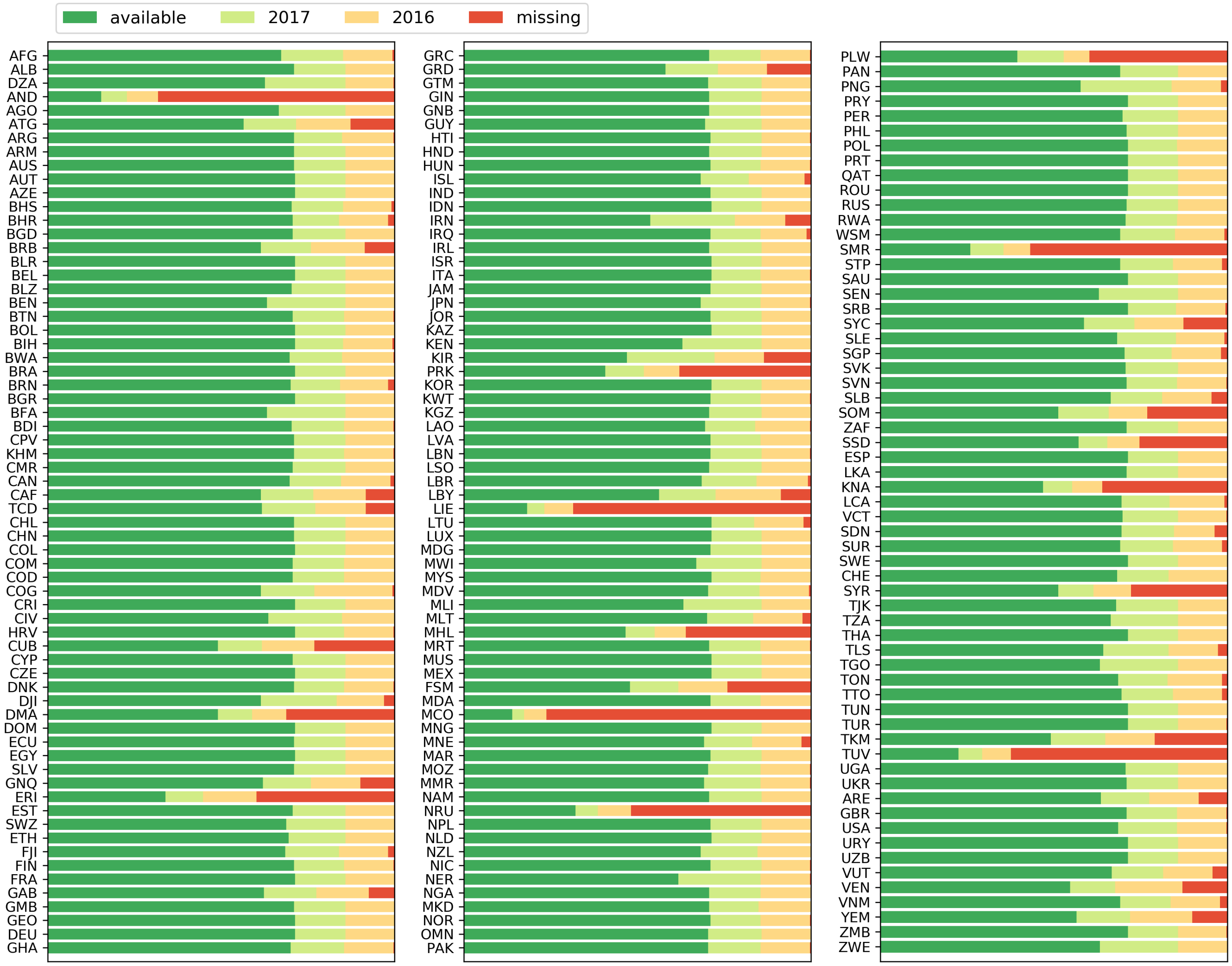

Figure S1. Indicator availability for each UN state for the 2018 data set. Bars of different colors represent the fraction of indicators with 2018 values available (green), indicators with values borrowed from 2017 (light green) and 2016 (orange), indicators with missing values for the period 2016–2018 (red).

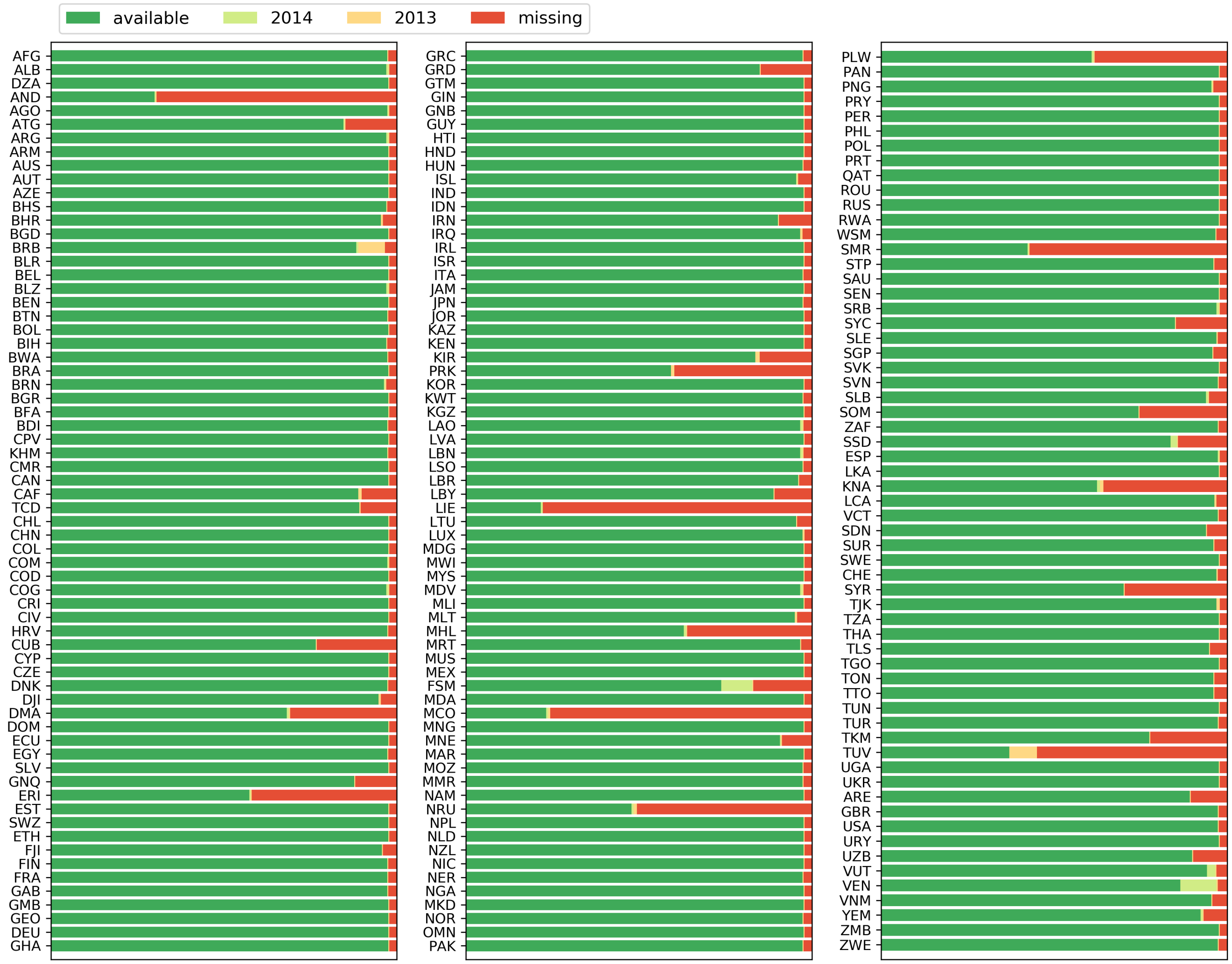

Figure S2. Indicator availability for each UN state for the 2015 data set. Bars of different colors represent the fraction of indicators with 2015 values available (green), indicators with values borrowed from 2014 (light green) and 2013 (orange), indicators with missing values for the period 2013–2015 (red).

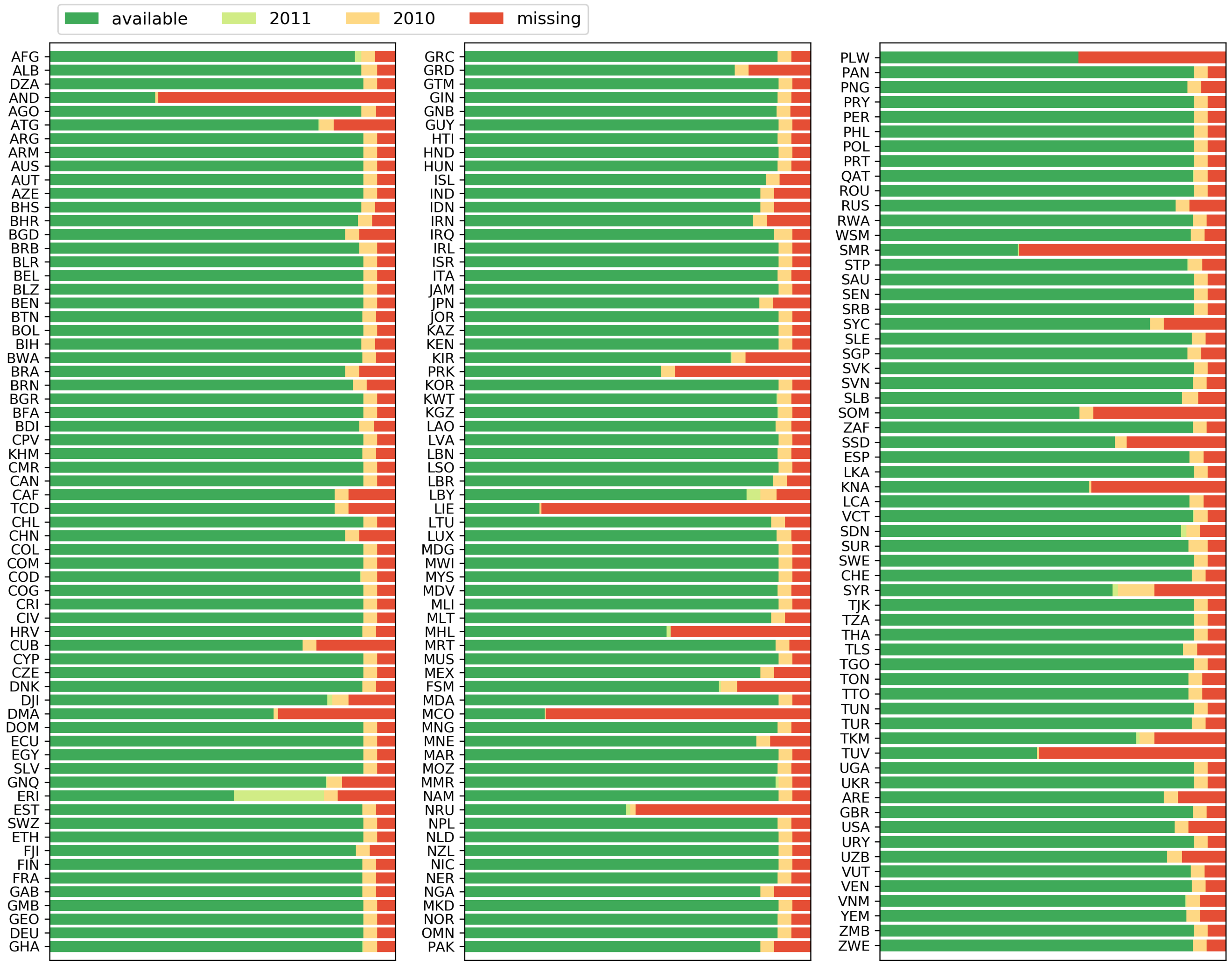

Figure S3. Indicator availability for each UN state for the 2012 data set. Bars of different colors represent the fraction of indicators with 2012 values available (green), indicators with values borrowed from 2011 (light green) and 2010 (orange), indicators with missing values for the period 2010–2012 (red).

S4 Development similarities of UN states

Table S8 reports the most and least correlated nodes in the 2018 WDI network, as well as the average correlation (link weight), for each UN country. Figure S4 reports the full set of correlations in the 2018 WDI network, represented as a heat map.

Table S8. The table reports, for each UN country, the most and least similar country in terms of correlation in the 2018 WDI network, along with the average correlation with other countries.

Country	Most similar country (correlation)	Least similar country (correlation)	Average correlation
Afghanistan	Cameroon (0.7200)	United States (-0.2159)	0.3409
Albania	Serbia (0.8302)	South Sudan (0.0303)	0.5506
Algeria	Tunisia (0.7697)	United States (0.0289)	0.4729
Andorra	Portugal (0.9031)	South Sudan (-0.2278)	0.4917
Angola	Benin (0.7750)	United States (-0.2195)	0.3715
Antigua and Barbuda	St. Kitts and Nevis (0.8878)	South Sudan (0.0876)	0.5787
Argentina	Uruguay (0.8111)	Somalia (-0.0389)	0.4934
Armenia	Georgia (0.8326)	South Sudan (0.0372)	0.5141
Australia	Canada (0.8798)	Somalia (-0.2737)	0.4096
Austria	Sweden (0.9105)	Somalia (-0.2267)	0.4835
Azerbaijan	Albania (0.7667)	Somalia (0.0375)	0.5184
Bahamas, The	Antigua and Barbuda (0.8455)	Somalia (0.0481)	0.5466
Bahrain	United Arab Emirates (0.8384)	South Sudan (-0.0268)	0.4724
Bangladesh	Myanmar (0.7798)	United States (-0.0549)	0.4584
Barbados	Grenada (0.8549)	Somalia (-0.0365)	0.5281
Belarus	Lithuania (0.8747)	Somalia (-0.1540)	0.5061
Belgium	Portugal (0.8856)	Somalia (-0.2236)	0.4481
Belize	Cabo Verde (0.8467)	United States (0.1658)	0.5840
Benin	Togo (0.8769)	United States (-0.2081)	0.4273
Bhutan	Nepal (0.7411)	United States (0.0262)	0.5153
Bolivia	Ecuador (0.8407)	United States (0.0686)	0.5398
Bosnia and Herzegovina	Serbia (0.8870)	South Sudan (-0.0812)	0.5093
Botswana	Namibia (0.8499)	United States (0.1130)	0.5593
Brazil	Colombia (0.7268)	Somalia (-0.1685)	0.4276
Brunei Darussalam	Malaysia (0.7252)	Somalia (-0.0789)	0.4720
Bulgaria	Romania (0.9111)	Somalia (-0.1750)	0.5065
Burkina Faso	Mali (0.8622)	United States (-0.2508)	0.3842
Burundi	Uganda (0.8618)	United States (-0.2171)	0.3504
Cabo Verde	Belize (0.8467)	United States (0.1191)	0.6080
Cambodia	Lao PDR (0.8298)	United States (0.0069)	0.5113
Cameroon	Tanzania (0.8242)	United States (-0.1809)	0.4301
Canada	Australia (0.8798)	Somalia (-0.3012)	0.4088
Central African Republic	Chad (0.8508)	United States (-0.3158)	0.2859
Chad	Niger (0.8734)	United States (-0.3935)	0.2216
Chile	Costa Rica (0.8498)	Somalia (-0.0979)	0.5613
China	Russian Federation (0.5719)	Somalia (-0.2680)	0.2090
Colombia	Costa Rica (0.9091)	Somalia (0.0176)	0.5985
Comoros	Kenya (0.7473)	United States (-0.1548)	0.4317
Congo, Dem. Rep.	Cameroon (0.7398)	United States (-0.3830)	0.2293
Congo, Rep.	Gabon (0.7575)	United States (-0.2847)	0.3623
Costa Rica	Colombia (0.9091)	Somalia (-0.0206)	0.5893
Cote d’Ivoire	Benin (0.8382)	United States (-0.1684)	0.4455
Croatia	Serbia (0.9151)	Somalia (-0.1609)	0.5210
Cuba	Costa Rica (0.8526)	South Sudan (-0.1134)	0.5405
Cyprus	Israel (0.8644)	Somalia (-0.1376)	0.5292
Czech Republic	Slovak Republic (0.9439)	Somalia (-0.2087)	0.5125
Denmark	Finland (0.9141)	Somalia (-0.2101)	0.4645
Djibouti	Benin (0.6577)	United States (-0.1163)	0.4488

Dominica	St. Vincent and the Grenadines (0.8824)	United States (0.0657)	0.5541
Dominican Republic	Ecuador (0.8609)	South Sudan (0.0782)	0.5958
Ecuador	Paraguay (0.8871)	Somalia (0.1453)	0.5955
Egypt, Arab Rep.	Jordan (0.7800)	United States (-0.0026)	0.4847
El Salvador	Guatemala (0.8973)	South Sudan (0.1369)	0.5720
Equatorial Guinea	Congo, Rep. (0.7299)	United States (-0.2530)	0.3147
Eritrea	Niger (0.6840)	United States (-0.2772)	0.2678
Estonia	Latvia (0.9178)	Somalia (-0.2180)	0.5090
Eswatini	Botswana (0.8065)	United States (-0.0099)	0.4538
Ethiopia	Uganda (0.8272)	United States (-0.2038)	0.3638
Fiji	Tonga (0.8448)	United States (0.1201)	0.5868
Finland	Sweden (0.9555)	Somalia (-0.2235)	0.4692
France	Germany (0.8635)	South Sudan (-0.2932)	0.3515
Gabon	Congo, Rep. (0.7575)	United States (-0.1120)	0.4199
Gambia, The	Senegal (0.8463)	United States (-0.1715)	0.4493
Georgia	Armenia (0.8326)	South Sudan (0.0318)	0.5321
Germany	France (0.8635)	Somalia (-0.3287)	0.3126
Ghana	Senegal (0.8021)	United States (-0.0853)	0.5204
Greece	Spain (0.8790)	South Sudan (-0.2071)	0.4657
Grenada	St. Lucia (0.9047)	South Sudan (0.0591)	0.5656
Guatemala	El Salvador (0.8973)	United States (0.1385)	0.5630
Guinea	Mali (0.8408)	United States (-0.3038)	0.3467
Guinea-Bissau	Benin (0.8213)	United States (-0.2336)	0.3761
Guyana	Suriname (0.7624)	United States (0.0541)	0.5339
Haiti	Gambia, The (0.6460)	United States (-0.2145)	0.3696
Honduras	Nicaragua (0.8883)	United States (0.1239)	0.5595
Hungary	Slovak Republic (0.9100)	Somalia (-0.2067)	0.4931
Iceland	Norway (0.9193)	Somalia (-0.1586)	0.5006
India	Pakistan (0.6573)	Andorra (-0.0659)	0.2395
Indonesia	Malaysia (0.7612)	Somalia (0.0840)	0.5000
Iran, Islamic Rep.	Tunisia (0.8227)	South Sudan (0.0131)	0.4752
Iraq	Libya (0.7353)	United States (-0.0909)	0.4029
Ireland	Andorra (0.8366)	Somalia (-0.1988)	0.4340
Israel	Iceland (0.8678)	Somalia (-0.0343)	0.5729
Italy	Spain (0.9207)	South Sudan (-0.3064)	0.4096
Jamaica	Costa Rica (0.8746)	Somalia (0.0580)	0.6012
Japan	Germany (0.8588)	Somalia (-0.3081)	0.3102
Jordan	Lebanon (0.8329)	South Sudan (0.0707)	0.5253
Kazakhstan	Kyrgyz Republic (0.7949)	Somalia (-0.0228)	0.5417
Kenya	Uganda (0.7986)	United States (-0.0909)	0.4736
Kiribati	Tuvalu (0.8857)	United States (-0.0252)	0.4759
Korea, Dem. Peoples Rep.	Myanmar (0.6998)	Nigeria (0.0962)	0.4174
Korea, Rep.	Singapore (0.8065)	Somalia (-0.2317)	0.4711
Kuwait	United Arab Emirates (0.8268)	Somalia (-0.0495)	0.4411
Kyrgyz Republic	Uzbekistan (0.8330)	United States (0.0656)	0.5526
Lao PDR	Cambodia (0.8298)	United States (-0.0630)	0.4719
Latvia	Lithuania (0.9461)	Somalia (-0.1807)	0.5280
Lebanon	Jordan (0.8329)	South Sudan (0.0466)	0.5368
Lesotho	Eswatini (0.7630)	United States (-0.1619)	0.3928
Liberia	Sierra Leone (0.7168)	United States (-0.2294)	0.3324
Libya	Algeria (0.7507)	United States (-0.0235)	0.4364
Liechtenstein	Switzerland (0.8216)	Congo, Dem. Rep. (-0.1245)	0.4966
Lithuania	Latvia (0.9461)	Somalia (-0.2196)	0.4964
Luxembourg	Monaco (0.8830)	Somalia (-0.2158)	0.4121
Madagascar	Tanzania (0.8332)	United States (-0.1372)	0.4046
Malawi	Uganda (0.8598)	United States (-0.1095)	0.4342

Malaysia	Costa Rica (0.8397)	Somalia (-0.0095)	0.5814
Maldives	St. Kitts and Nevis (0.7436)	South Sudan (0.0031)	0.4788
Mali	Niger (0.8767)	United States (-0.2525)	0.3807
Malta	Andorra (0.8459)	Somalia (-0.1644)	0.4647
Marshall Islands	Tuvalu (0.8730)	United States (0.1199)	0.5214
Mauritania	Senegal (0.7796)	United States (-0.2587)	0.3929
Mauritius	Sri Lanka (0.8058)	South Sudan (-0.0446)	0.5700
Mexico	Colombia (0.8451)	South Sudan (-0.0244)	0.5419
Micronesia, Fed. Sts.	Tuvalu (0.8047)	United States (0.0332)	0.4595
Moldova	Romania (0.8172)	Somalia (-0.0694)	0.4989
Monaco	Luxembourg (0.8830)	South Sudan (-0.2905)	0.5124
Mongolia	Kyrgyz Republic (0.7367)	United States (-0.0242)	0.4741
Montenegro	Bosnia and Herzegovina (0.8782)	South Sudan (-0.0934)	0.5304
Morocco	Tunisia (0.8468)	United States (0.1141)	0.5630
Mozambique	Malawi (0.8352)	United States (-0.1680)	0.3837
Myanmar	Bangladesh (0.7798)	United States (-0.0785)	0.4761
Namibia	Botswana (0.8499)	United States (-0.0141)	0.4762
Nauru	Tuvalu (0.7870)	United States (0.1051)	0.5972
Nepal	Lao PDR (0.7723)	United States (-0.0300)	0.4834
Netherlands	Switzerland (0.8914)	Somalia (-0.2383)	0.4183
New Zealand	Iceland (0.9024)	Somalia (-0.1911)	0.5140
Nicaragua	Honduras (0.8883)	United States (0.1440)	0.5530
Niger	Mali (0.8767)	United States (-0.2599)	0.3284
Nigeria	Cameroon (0.7312)	United States (-0.2427)	0.2912
North Macedonia	Serbia (0.8946)	Chad (-0.0416)	0.5468
Norway	Sweden (0.9476)	Somalia (-0.2295)	0.4481
Oman	United Arab Emirates (0.8450)	Somalia (0.0169)	0.4336
Pakistan	Bangladesh (0.7793)	United States (-0.1023)	0.4063
Palau	Fiji (0.8364)	South Sudan (0.0978)	0.5716
Panama	Colombia (0.8646)	Somalia (0.0125)	0.5699
Papua New Guinea	Timor-Leste (0.7139)	United States (-0.1294)	0.3918
Paraguay	Ecuador (0.8871)	Somalia (0.1579)	0.5762
Peru	Ecuador (0.8823)	Somalia (0.0635)	0.5745
Philippines	Indonesia (0.7453)	United States (0.1298)	0.5296
Poland	Slovak Republic (0.9055)	Somalia (-0.1936)	0.5264
Portugal	Spain (0.9156)	Somalia (-0.2429)	0.4869
Qatar	United Arab Emirates (0.8738)	Somalia (-0.0689)	0.4170
Romania	Czech Republic (0.9223)	Somalia (-0.1386)	0.5362
Russian Federation	Kazakhstan (0.7085)	Somalia (-0.2990)	0.3752
Rwanda	Uganda (0.8259)	United States (-0.0424)	0.4634
Samoa	Tonga (0.7164)	Eritrea (0.0123)	0.4615
San Marino	Monaco (0.8670)	Congo, Dem. Rep. (-0.1933)	0.4973
Sao Tome and Principe	Cabo Verde (0.7912)	United States (-0.0670)	0.4989
Saudi Arabia	Kuwait (0.7819)	Chad (0.0078)	0.4524
Senegal	Gambia, The (0.8463)	United States (-0.1339)	0.4891
Serbia	Croatia (0.9151)	Somalia (-0.0767)	0.5378
Seychelles	Antigua and Barbuda (0.8060)	South Sudan (0.0028)	0.5505
Sierra Leone	Guinea (0.7697)	United States (-0.2447)	0.3283
Singapore	Korea, Rep. (0.8065)	Somalia (-0.1938)	0.4363
Slovak Republic	Czech Republic (0.9439)	Somalia (-0.1656)	0.5341
Slovenia	Czech Republic (0.9064)	Somalia (-0.2098)	0.5038
Solomon Islands	Vanuatu (0.8248)	United States (-0.0659)	0.4395
Somalia	Central African Republic (0.7553)	United States (-0.3893)	0.1674
South Africa	Namibia (0.8171)	Eritrea (0.0765)	0.4898
South Sudan	Central African Republic (0.7581)	United States (-0.3691)	0.1671
Spain	Italy (0.9207)	South Sudan (-0.2845)	0.4341
Sri Lanka	Mauritius (0.8058)	South Sudan (-0.0166)	0.5438

St. Kitts and Nevis	Antigua and Barbuda (0.8878)	South Sudan (0.0988)	0.5632
St. Lucia	Grenada (0.9047)	Somalia (0.0519)	0.5467
St. Vincent and the Grenadines	Dominica (0.8824)	Chad (0.0858)	0.5406
Sudan	Yemen, Rep. (0.8159)	United States (-0.2709)	0.3533
Suriname	Guyana (0.7624)	South Sudan (0.0841)	0.5039
Sweden	Finland (0.9555)	Somalia (-0.2195)	0.4673
Switzerland	Austria (0.9068)	Somalia (-0.2288)	0.4344
Syrian Arab Republic	Iraq (0.7137)	United States (-0.0547)	0.3127
Tajikistan	Kyrgyz Republic (0.7991)	United States (-0.0566)	0.4913
Tanzania	Malawi (0.8332)	United States (-0.1672)	0.3997
Thailand	Malaysia (0.8142)	Somalia (-0.0438)	0.5625
Timor-Leste	Papua New Guinea (0.7139)	United States (-0.1496)	0.4166
Togo	Benin (0.8769)	United States (-0.2077)	0.4224
Tonga	Fiji (0.8448)	United States (0.1405)	0.5533
Trinidad and Tobago	Barbados (0.8056)	Somalia (-0.0507)	0.5465
Tunisia	Morocco (0.8468)	South Sudan (0.0427)	0.5494
Turkey	Tunisia (0.8109)	South Sudan (-0.0043)	0.5417
Turkmenistan	Uzbekistan (0.8302)	United States (0.1129)	0.5267
Tuvalu	Kiribati (0.8857)	China (0.1955)	0.5804
Uganda	Burundi (0.8618)	United States (-0.1969)	0.3906
Ukraine	Belarus (0.8317)	Somalia (-0.0885)	0.4821
United Arab Emirates	Qatar (0.8738)	Somalia (-0.1095)	0.4145
United Kingdom	France (0.8531)	Somalia (-0.2677)	0.3768
United States	United Kingdom (0.7111)	Chad (-0.3935)	0.1334
Uruguay	Costa Rica (0.8256)	Somalia (-0.0815)	0.5189
Uzbekistan	Kyrgyz Republic (0.8330)	United States (0.0396)	0.5157
Vanuatu	Solomon Islands (0.8248)	United States (0.0490)	0.5285
Venezuela, RB	Ecuador (0.6415)	India (-0.0156)	0.3756
Vietnam	Thailand (0.7847)	Somalia (0.0310)	0.5337
Yemen, Rep.	Sudan (0.8159)	United States (-0.2579)	0.3386
Zambia	Malawi (0.8331)	United States (-0.1478)	0.4256
Zimbabwe	Malawi (0.8393)	United States (-0.0532)	0.4506

S5 Community detection analysis

In this section, we discuss the choice of the parameters of the employed community detection algorithms. This selection was determined by the request of output consistency and robustness with respect to parameter variations. We analyze the performance of the community detection algorithms by representing the behavior with varying parameters of three quantities:

- the *percentage of agreement*, computed, for a given set of parameters, as the ratio (expressed in %) between the number of occurrences of the most common network partition and the total number of runs of the algorithm;
- the *number of communities* in the most common partition;
- the *inverse participation ratio* (IPR) in the most common partition, defined, for a partition in K subsets of a network with N nodes, as

$$\mathrm{IPR} = \frac{1}{\sum_{i=1}^{K} \left(\frac{n_i}{N}\right)^2}, \tag{S1}$$

with $(n_1, n_2, \ldots, n_K)$ the cardinalities of each subset. The IPR is a mathematical tool to evaluate the number of communities among which the considered network is "effectively" shared, discarding communities with a very small quantity of nodes; for example, a partition in $K = 3$ communities of a network of $N = 90$ nodes is characterized by $\mathrm{IPR} = 3$ if $n_1 = n_2 = n_3 = 30$, while a partition with cardinalities $(n_1 = 45, n_2 = 44, n_3 = 1)$ yields $\mathrm{IPR} = 2.044$, very close to 2.

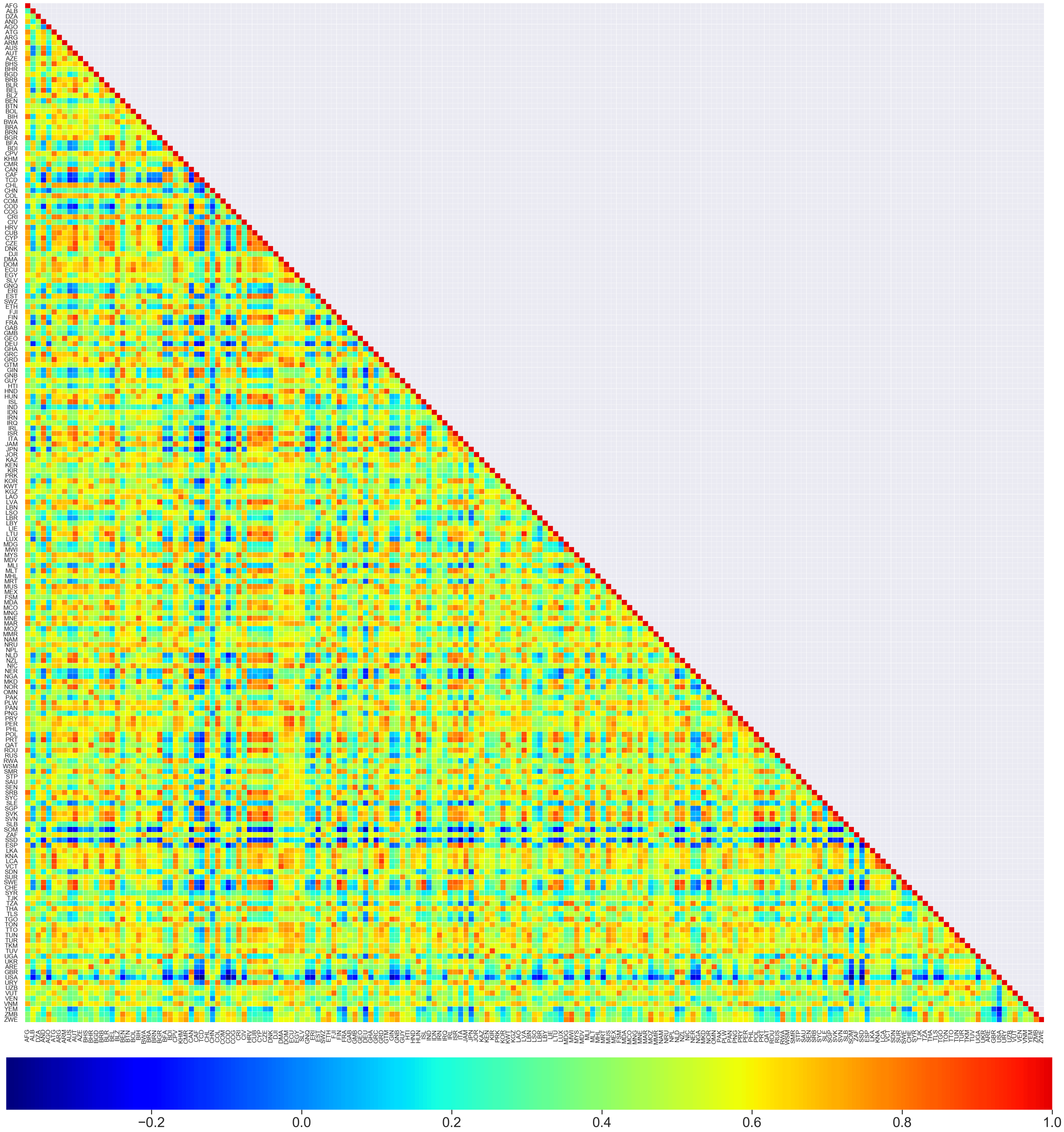

Figure S4. Heat map of correlations in the 2018 WDI network between the 193 UN countries, computed from the 324 selected WDI indicators.

Spin Glass algorithm

We start by analyzing the effects of parameter changes on the Spin Glass community detection algorithm, based on statistical mechanics[4,5]. Here, the output is determined by the resolution γ and the cooling factor. Figure S5 represents, in the context of hierarchical community detection, the features of network partition at level 1 (first row) for the whole network, and at level 2 for the two macrocommunities α (second row) and ω (third row) found at the previous stage. The first column shows, as a function of the resolution γ and the cooling factor, the percentage of agreement for the most common partition. The second

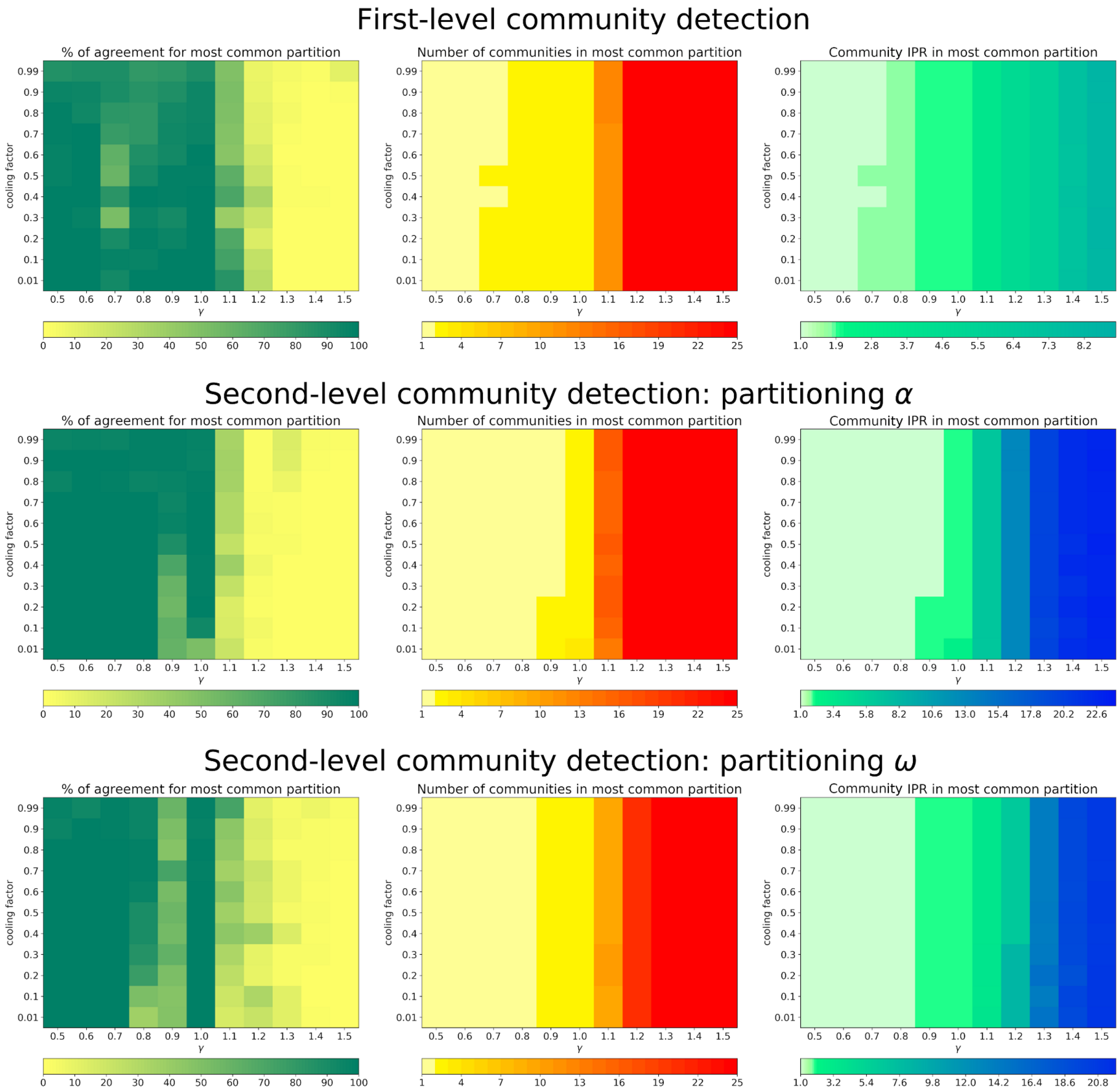

Figure S5. Results of the first and second level of hierarchical community detection with the Spin Glass algorithm, obtained with varying resolution γ and cooling factor.

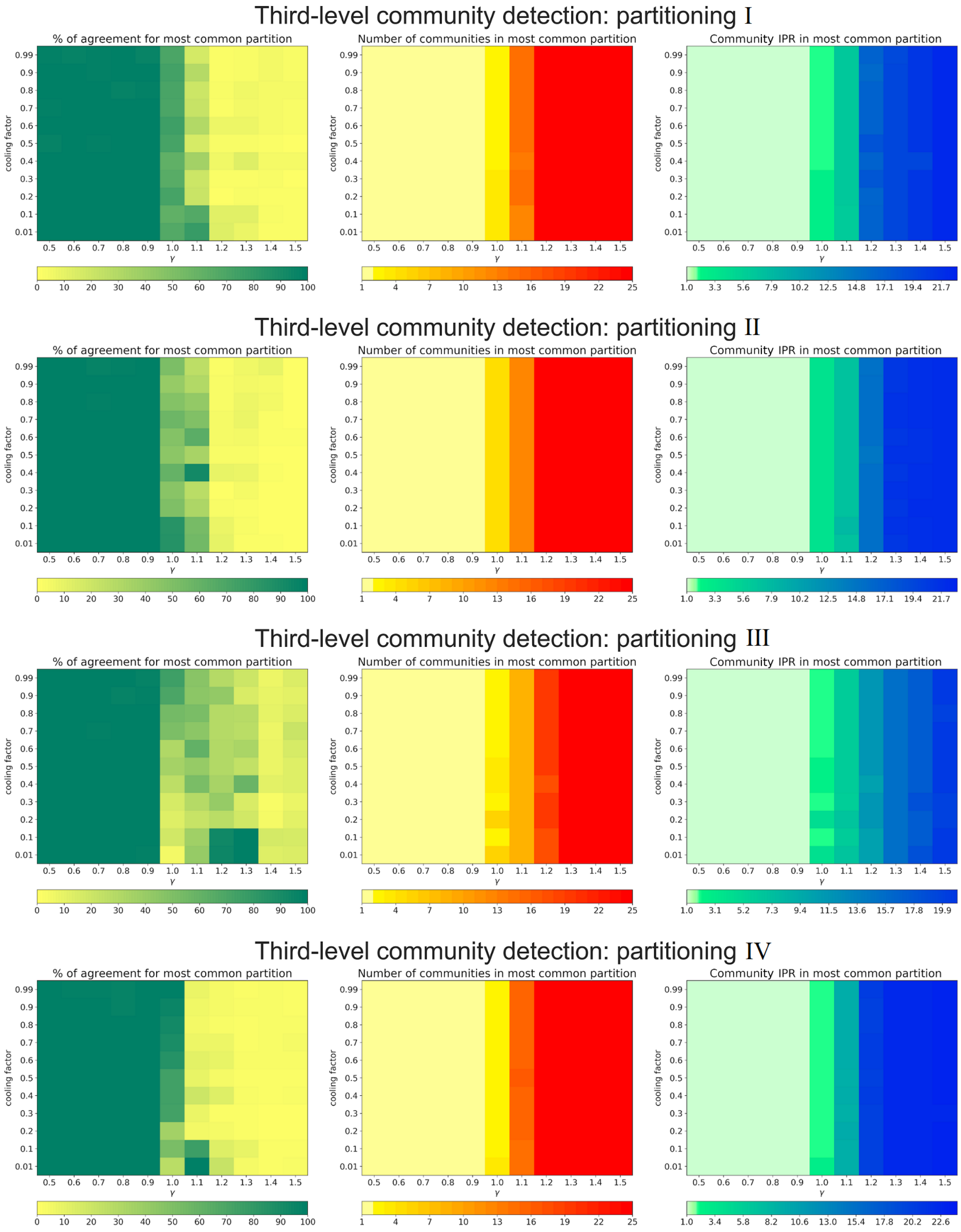

Figure S6. Results of the third level of hierarchical community detection with the Spin Glass algorithm, obtained with varying resolution γ and cooling factor.

column reports the number of communities found for each pair of aforementioned parameters in the most common partition, while the third column contains the corresponding IPR measures.

Considering the plots related to the search for macrocommunities in the whole network (first row in Fig. S5), we notice the existence of essentially two regions in which the algorithm output is stable, yielding more than 90% of agreement for the most common partition *independent of the cooling factor*. The first region corresponds to very low resolution values, in which the network is seen in the vast majority of runs as a single community, as one can observe from the plots of both number of communities and IPR in the most common partition. The second part of the parameter space in which the output is stable is determined by $0.8 \lesssim \gamma \lesssim 1$. For these intermediate resolutions, the Spin Glass algorithm finds the same partition almost unanimously, and from the plot in the second column one can get the information that such most common partition is made of two communities. These two parts of the network consist of different numbers of nodes, as proved by the IPR value, that is close, but not equal, to 2. Analyzing the values of IPR, we found that the most balanced communities, corresponding to $\mathrm{IPR} = 1.988$, were obtained for $\gamma = 1$, at any value of the cooling factor. The aforementioned regions are separated by an instability interval in which the partition in two communities is not robust, as it is evident from the light-green and yellow spots around $\gamma \simeq 0.7$ in the first-row percentage agreement map in Fig. S5. Furthermore, for $\gamma > 1.0$ the number of communities increases, and the accordance between different outputs tends to become very low. Notice that the number of communities switches to 3 as soon as the resolution reaches 1.01 (not shown in Fig. S5), and that for higher values of γ the number of communities becomes increasingly different from the IPR, signalling the presence of very small clusters.

Given the above considerations, we chose to fix the resolution γ for the macrocommunity detection in the whole network in the region $0.8 \lesssim \gamma \lesssim 1$, which gives nontrivial and stable results. Moreover, we selected the value $\gamma = 1.0$, which corresponds to the most balanced bipartition, involving 104 nodes in the macrocommunity α and 89 nodes in the macrocommunity ω, which includes countries with less favorable WDI indicators. The results do not depend crucially on the cooling factor, which was fixed at the value that maximized the agreement to the most common partition. The macrocommunities α and ω are both complete subgraphs of the initial network; α contains $104 * 103/2 = 5356$ edges, and the link between China and RB Venezuela is the least weighted (-0.004); ω contains $89 * 88/2 = 3916$ edges, with the link between Eritrea and Samoa being the least weighted (0.012).

Similar considerations apply for the second level of the hierarchical community detection (middle and bottom rows in Fig. S5), in which the two macrocommunities α and ω, found at the first stage, were partitioned in the communities (I,II) and (III,IV), respectively. Even in this case, the only configuration of both stable and nontrivial output is represented by a two-community network, and the value $\gamma = 1$ provides the most balanced division of both α and ω. Specifically, the macrocommunity α splits in community I, characterized on average by the most favorable WDI indicators, containing 46 nodes and $46 * 45/2 = 1035$ edges, with the link between Andorra and China being the least weighted (0.069), and community II, containing 58 nodes and $58 * 57/2 = 1653$ links, with the link between Brazil and Liechtenstein being the least weighted (0.261). The macrocommunity ω splits in community III, containing 45 nodes and $45 * 44/2 = 990$ edges, with the link between Dem. People's Rep. Korea and Samoa being the least weighted (0.159), and community IV, characterized on average by the least favorable WDI indicators, containing 44 nodes and $44 * 43/2 = 946$ links, with the link between Eritrea and Nigeria being the least weighted (0.315).

In Fig. S6, we show the results obtained when trying to partition the communities I, II, III and IV. At this level no community detection satisfies the criteria of being at the same time non trivial, stable and independent of the cooling factor. Therefore, we chose to stop the process of hierarchical detection to the second stage, in which we found four communities of comparable size.

Leiden algorithm

The subdivision in communities obtained from the hierarchical application of the Spin Glass algorithm has been substantially confirmed by the comparison with the Leiden algorithm[6], adapted to the presence of negative weights. We performed community detection with varying resolution γ and parameter β, which determines the level of randomness in the search for the optimal partition in the Leiden algorithm.

In general, as it can be observed from Figs. S7-S8, the Leiden algorithm tends to find an overall higher agreement on the most common partition. Another interesting observation is the fact that the features of the most common partition are almost entirely independent of β, at least in the considered range.

At of the first level of the hierarchical search, we observe, as reported in the upper panel of Fig. S7, that $\gamma = 1$ represents the largest resolution at which the algorithm provides with extremely large agreement a two-community partition, with $\mathrm{IPR} = 1.99$. As opposed to the case of the Spin Glass algorithm, here the agreement remains large and almost independent of β for $\gamma = 1.1$ and $\gamma = 1.2$, where the Leiden algorithm finds 11 communities with $\mathrm{IPR} = 3.54$ and 27 communities with $\mathrm{IPR} = 4.64$, respectively.

The situation changes at the second level. Here, for the macrocommunity α, the agreement drops significantly for $\gamma > 1.0$, to be recovered only for $\gamma = 1.3$, when the partition is highly fragmented; actually, even the agreement on the two-community

partition (IPR = 1.97) at $\gamma = 1$ tends to decrease by increasing β. Instead, the Leiden algorithm finds with full agreement, independent of β, a bipartition for the macrocommunity ω (IPR = 2.00) at $\gamma = 1$; two communities are found also for $\gamma = 0.9$, with a much smaller agreement (around 60%). Remarkably, the robustness of community detection is confirmed by the fact that both Spin Glass and Leiden algorithms find, at the first and second level of detection, identical partitions, leading to the same structure of four communities, discussed in the main text.

Community detection at the third level, whose results are represented in Fig. S8, always provides results with rather small agreements for $\gamma = 1$. However, while the most common partition found for the communities I, II and IV is constant with respect to β, this result changes for community III, where partitions in 2 or 3 communities can be found according to the value of β.

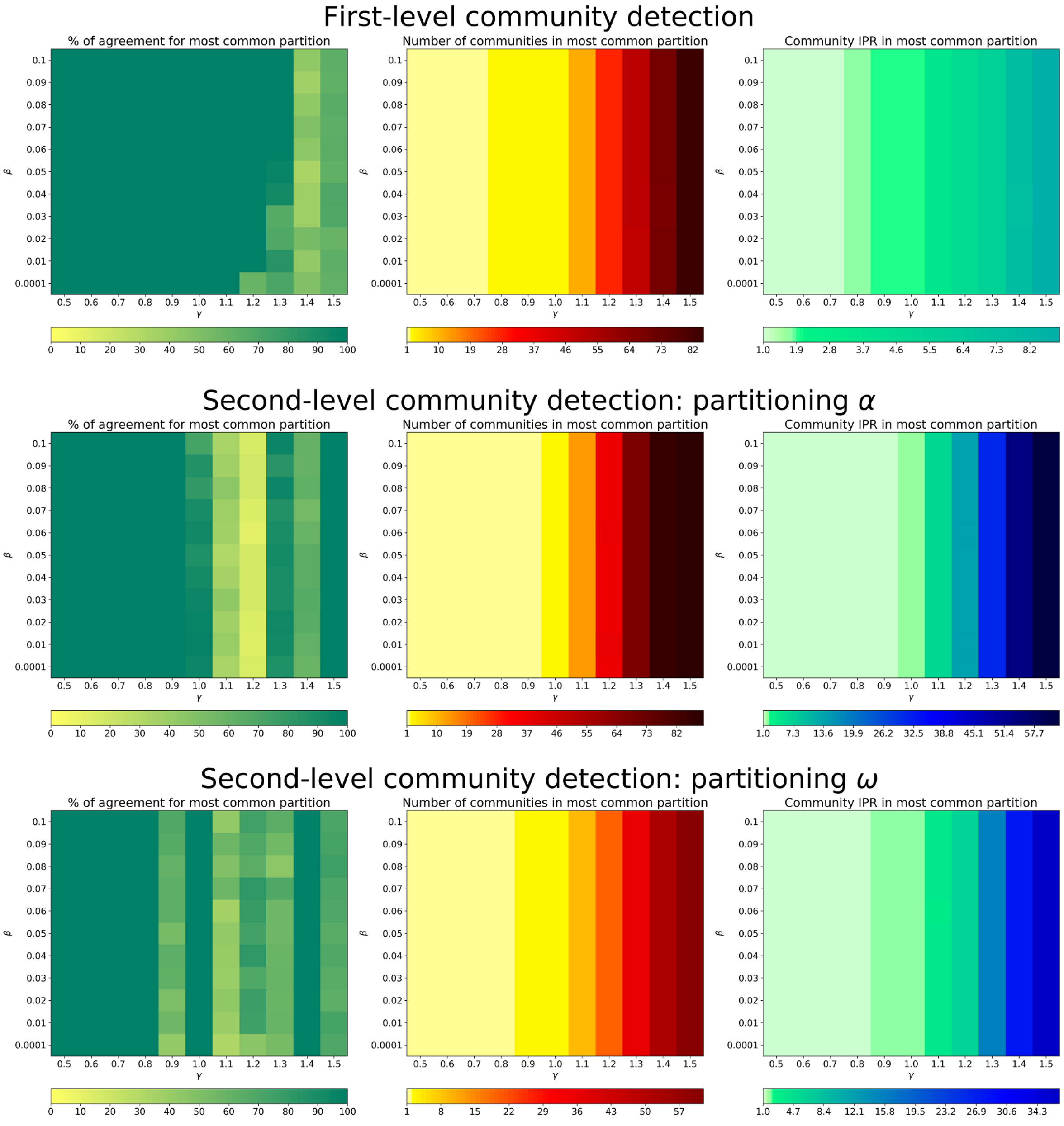

Figure S7. Results of the first and second level of hierarchical community detection with the Leiden algorithm, obtained with varying resolution γ and parameter β, determining the randomness of community search.

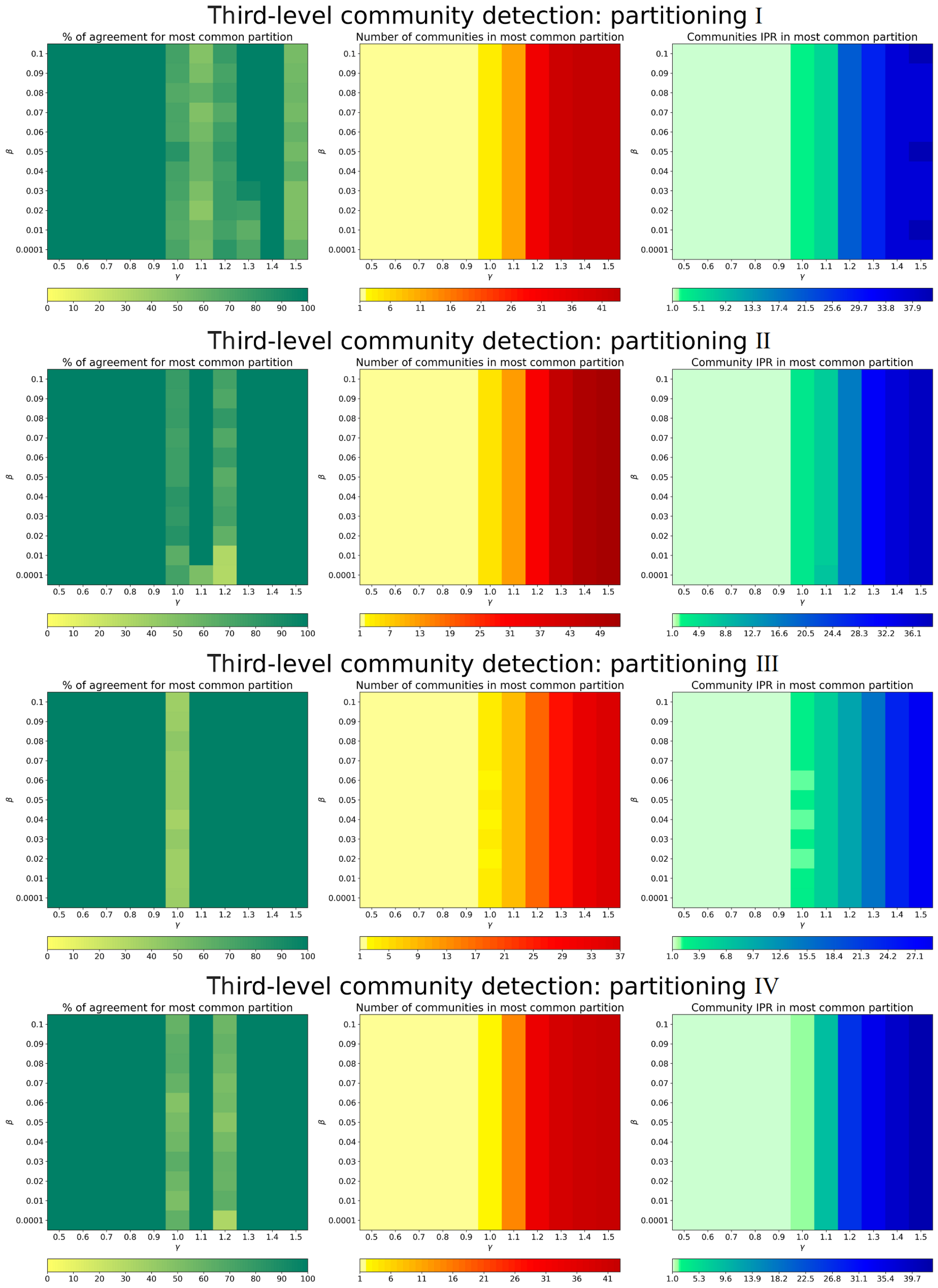

Figure S8. Results of the third level of hierarchical community detection with the Leiden algorithm, obtained with varying resolution γ and parameter β, determining the randomness of community search.

S6 Comparing communities with UN development groups and World Bank income groups

Our complex network model determines, through the algorithms examined in the previous section, a partition of UN member states in communities, based on the paradigm of similarity between WDIs. This partition is only partially overlapped to the ways of grouping countries employed by the UN, based on development, and by the World Bank, based on income[7]. However, we notice that the communities (I,II,III,IV) found in our analysis exhibit well-separated average development levels in a remarkable way, as one can observe from the results displayed in Fig. S9. This feature allows to sort communities in a hierarchical development ordering from I to IV, corroborating the idea to refer to them as *development communities*.

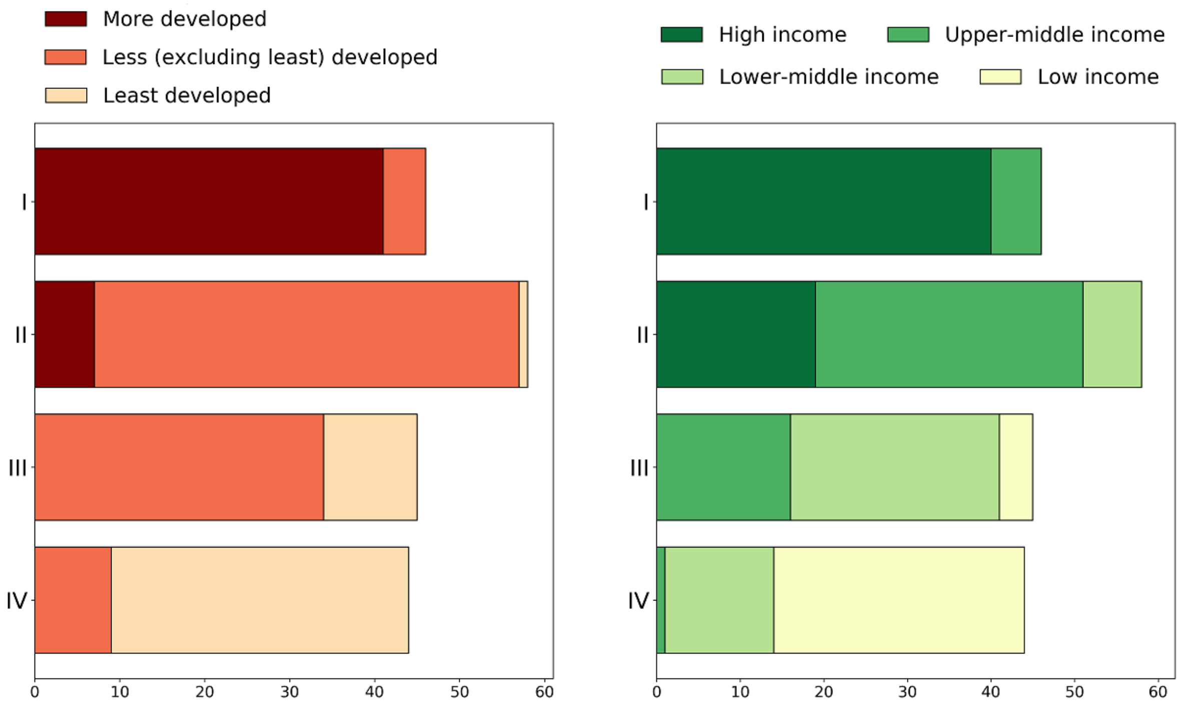

Figure S9. Composition of WDI network communities I, II, III and IV in terms of the three UN development groups (left) and the four World Bank income groups (right).

S7 Distributions of ranked indexes in communities (high-resolution figures)

For the sake of clarity, we show in Figs. S10–S14 the high-resolution versions of the distributions of the five considered rank indexes, as reported in Figs. 3-4 of the main text.

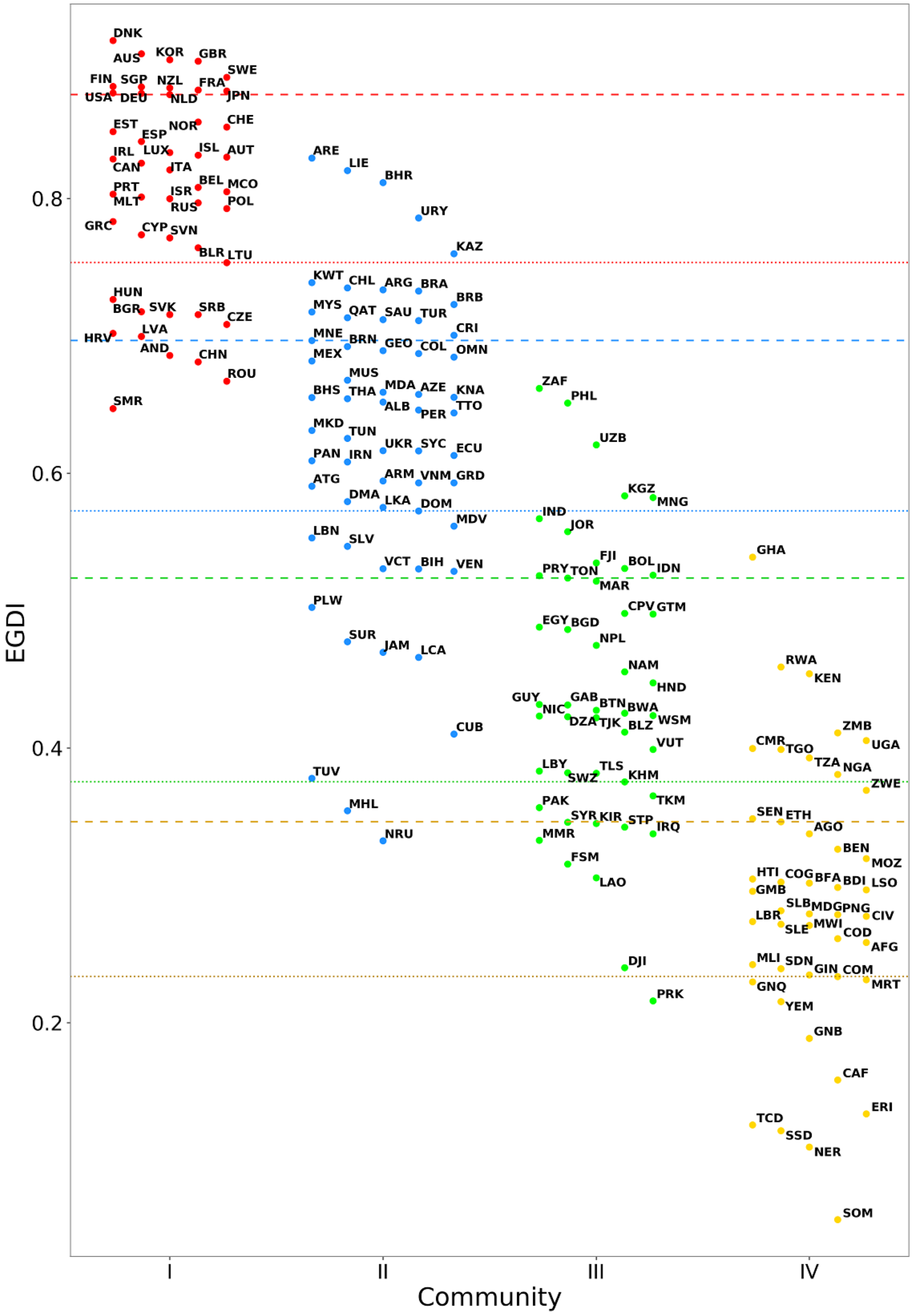

Figure S10. Distribution of EGDI of UN countries, separated in communities I (red), II (blue), III (green) and IV (yellow). Dashed and dotted lines of the same color as communities represent the 25th and 75th percentile, respectively, of the associated community distributions.

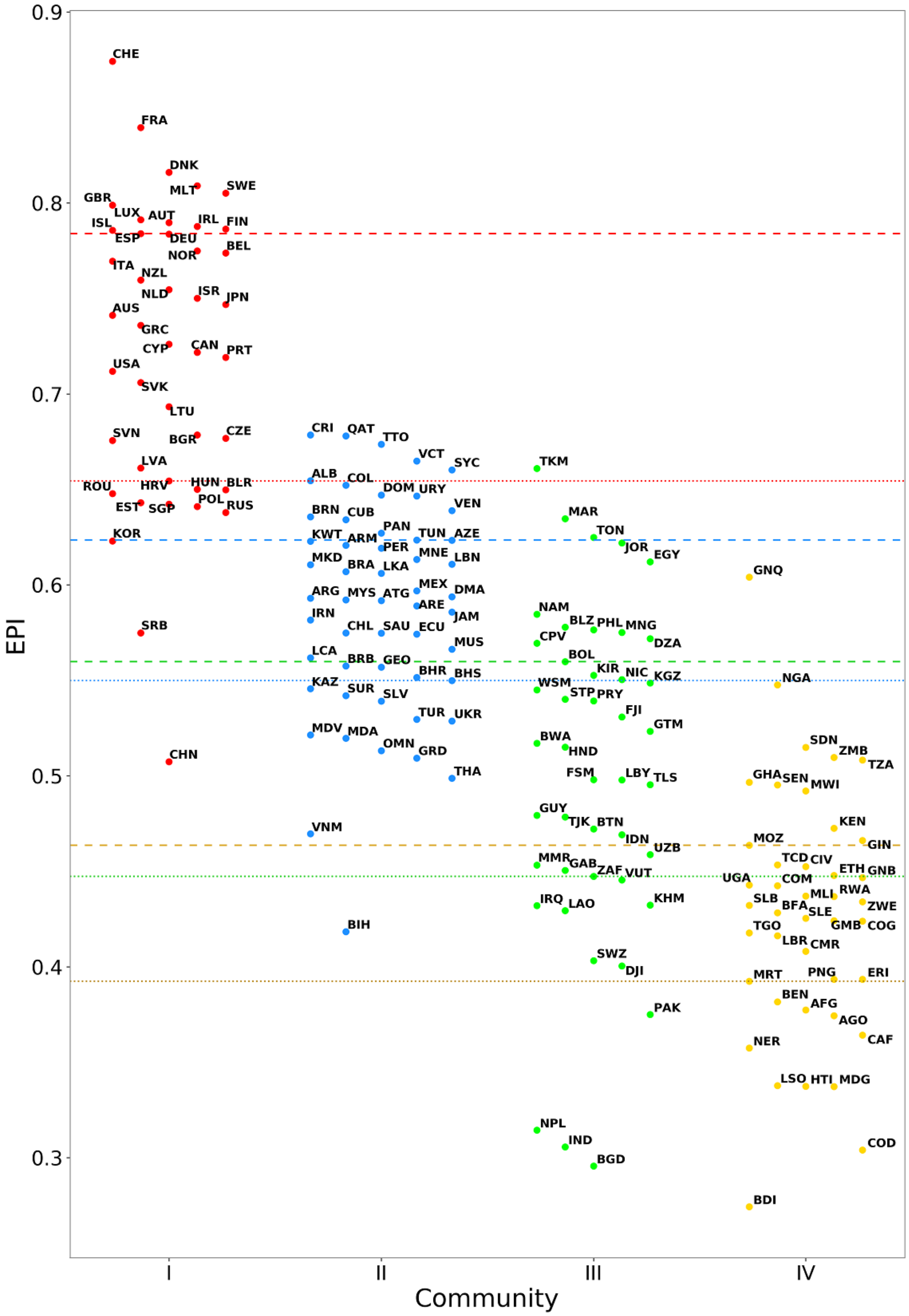

Figure S11. Distribution of EPI of UN countries, separated in communities I (red), II (blue), III (green) and IV (yellow). Dashed and dotted lines of the same color as communities represent the 25th and 75th percentile, respectively, of the associated community distributions.

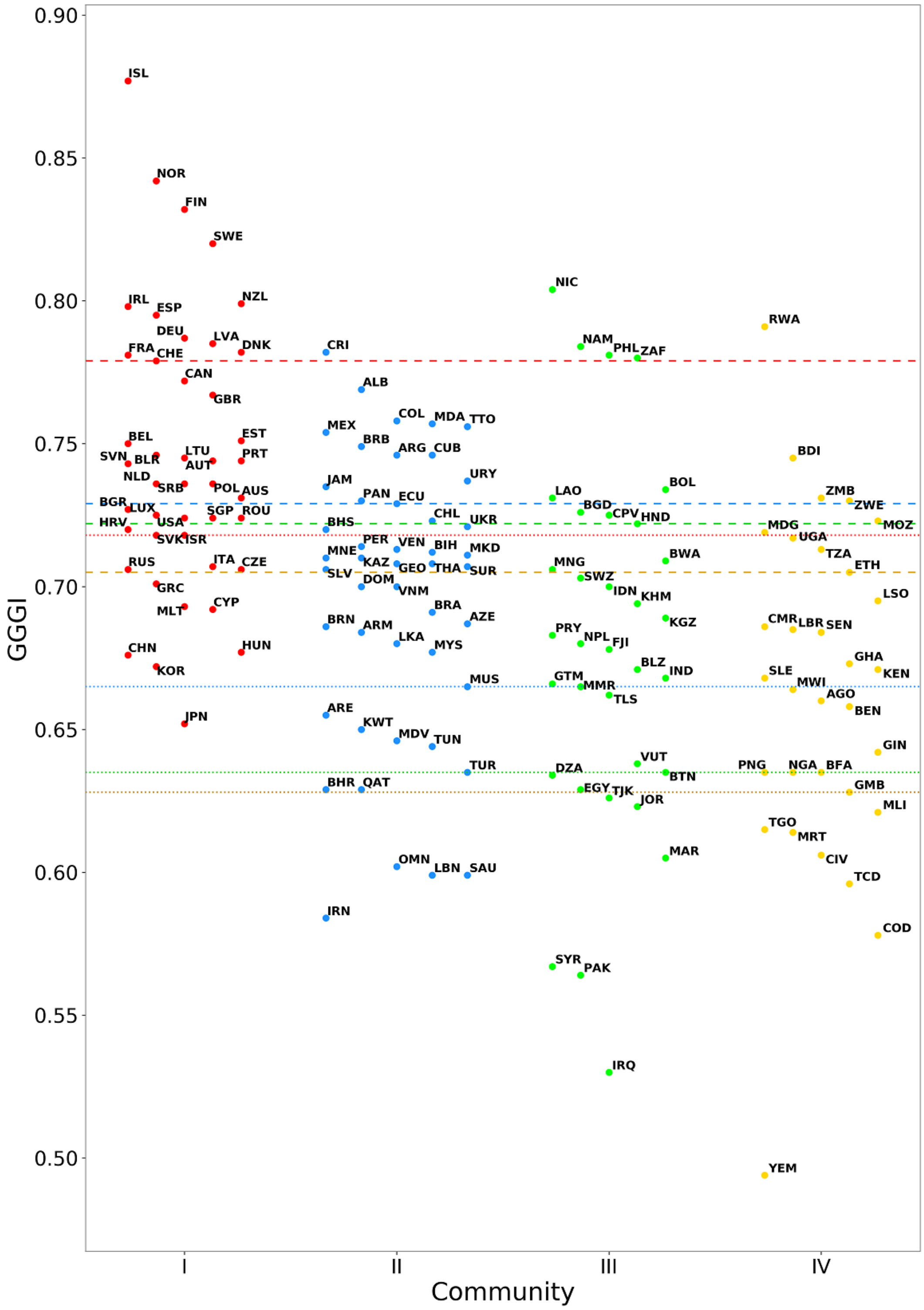

Figure S12. Distribution of GGGI of UN countries, separated in communities I (red), II (blue), III (green) and IV (yellow). Dashed and dotted lines of the same color as communities represent the 25th and 75th percentile, respectively, of the associated community distributions.

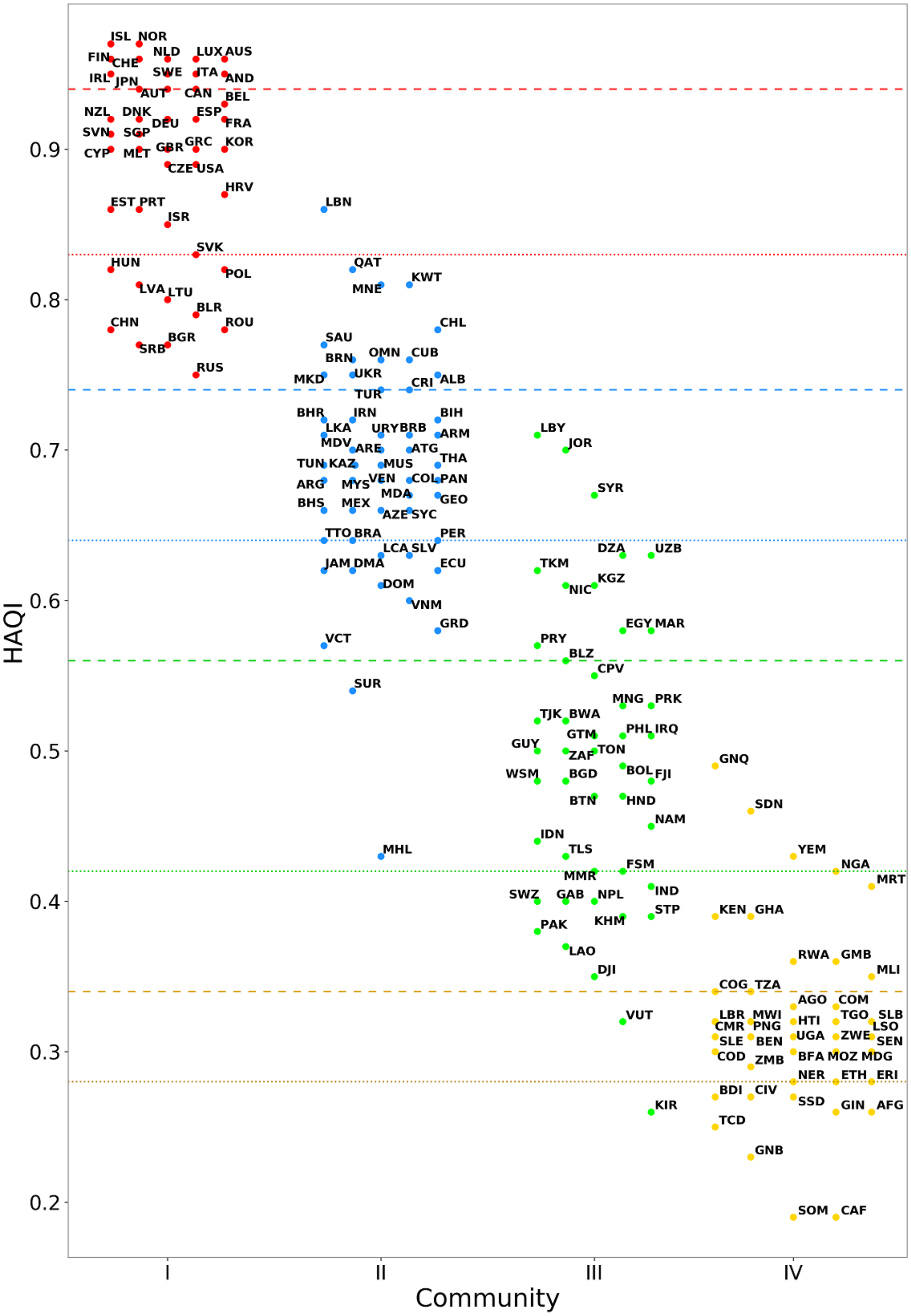

Figure S13. Distribution of HAQI of UN countries, separated in communities I (red), II (blue), III (green) and IV (yellow). Dashed and dotted lines of the same color as communities represent the 25th and 75th percentile, respectively, of the associated community distributions.

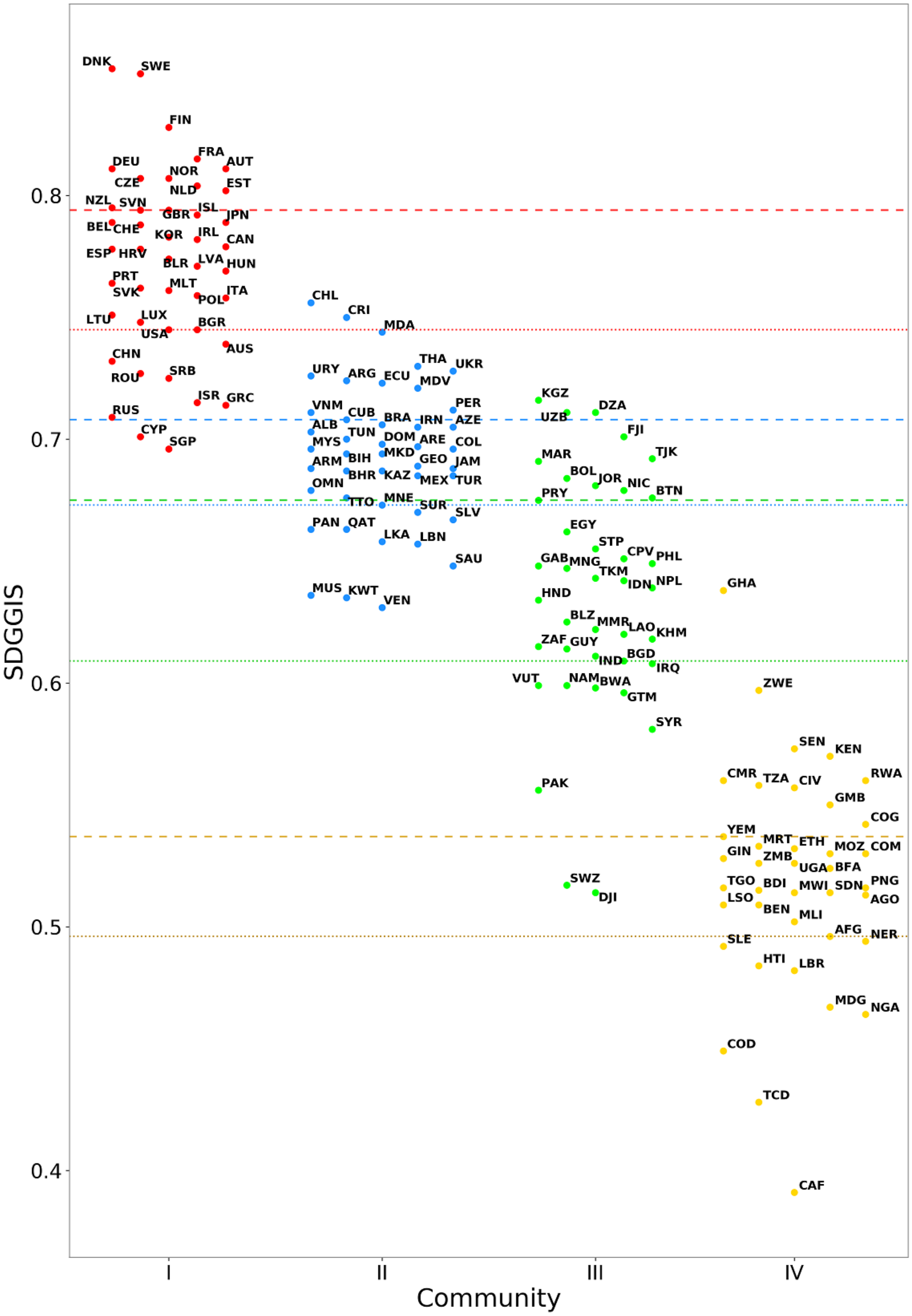

Figure S14. Distribution of SDGGIS of UN countries, separated in communities I (red), II (blue), III (green) and IV (yellow). Dashed and dotted lines of the same color as communities represent the 25th and 75th percentile, respectively, of the associated community distributions.

S8 Further applications of community-based rating scheme

In Tabs. S9–S11 we report the results of the community-based country classification, discussed in Sec. 2.3 of the main text, for the EPI[8,9], HAQI[10] and SDGGIS[11].

Table S9. Classification of countries based on the quartiles (Q_1, Q_2, Q_3, Q_4) of the overall EPI distribution and on the development community membership.

	I	II	III	IV
Q_1	**A**<br>CHE, FRA, DNK, MLT, SWE, GBR, LUX, AUT, IRL, FIN, ISL, ESP, DEU, NOR, BEL, ITA, NZL, NLD, ISR, JPN, AUS, GRC, CYP, CAN, PRT, USA, SVK, LTU, BGR, CZE, SVN, LVA, HRV, HUN, BLR, ROU	**Aa**<br>CRI, QAT, TTO, VCT, SYC, ALB, COL, DOM	**Aaa**<br>TKM	
Q_2	**B**<br>EST, SGP, POL, RUS, KOR, SRB	**Bb**<br>URY, VEN, BRN, CUB, PAN, TUN, AZE, KWT, ARM, PER, MNE, LBN, MKD, BRA, LKA, MEX, DMA, ARG, MYS, ATG, ARE, JAM, IRN, CHL, SAU, ECU, MUS	**Bbb**<br>MAR, TON, JOR, EGY, NAM, BLZ, PHL, MNG, DZA, CPV	**Bbbb**<br>GNQ
Q_3	**C**<br>CHN	**Cc**<br>LCA, BRB, GEO, BHR, BHS, KAZ, SUR, SLV, TUR, UKR, MDV, MDA, OMN, GRD, THA, VNM	**Ccc**<br>BOL, KIR, NIC, KGZ, WSM, STP, PRY, FJI, GTM, BWA, HND, FSM, LBY, TLS, GUY, TJK, BTN, IDN	**Cccc**<br>NGA, SDN, ZMB, TZA, GHA, SEN, MWI, KEN, GIN, MOZ
Q_4		**D**<br>BIH	**Dd**<br>UZB, MMR, GAB, ZAF, VUT, KHM, IRQ, LAO, SWZ, DJI, PAK, NPL, IND, BGD	**Ddd**<br>TCD, CIV, ETH, GNB, UGA, COM, MLI, RWA, ZWE, SLB, BFA, SLE, GMB, COG, TGO, LBR, CMR, PNG, ERI, MRT, BEN, AFG, AGO, CAF, NER, LSO, HTI, MDG, COD, BDI

Table S10. Classification of countries based on the quartiles (Q_1, Q_2, Q_3, Q_4) of the overall HAQI distribution and on the development community membership.

	I	II	III	IV
**Q1**	**A**<br>ISL, NOR, NLD, LUX, AUS, FIN, CHE, SWE, ITA, AND, IRL, JPN, AUT, CAN, BEL, NZL, DNK, DEU, ESP, FRA, SVN, SGP, GBR, GRC, KOR, CYP, MLT, CZE, USA, HRV, EST, PRT, ISR, SVK, POL, HUN, LVA, LTU, BLR, ROU, CHN	**Aa**<br>LBN, QAT, MNE, KWT, CHL		
**Q2**	**B**<br>SRB, BGR, RUS	**Bb**<br>SAU, BRN, OMN, CUB, ALB, MKD, UKR, TUR, CRI, BIH, BHR, IRN, URY, BRB, ARM, LKA, MDV, ARE, ATG, THA, TUN, KAZ, MUS, COL, PAN, ARG, MYS, VEN, MDA, GEO, BHS, MEX, AZE, SYC, PER, TTO, BRA, LCA, SLV	**Bbb**<br>LBY, JOR, SYR, DZA, UZB	
**Q3**		**C**<br>ECU, JAM, DMA, DOM, VNM, GRD, VCT, SUR, MHL	**Cc**<br>TKM, NIC, KGZ, EGY, MAR, PRY, BLZ, CPV, MNG, PRK, TJK, BWA, GTM, PHL, IRQ, GUY, ZAF, TON, BOL, FJI, WSM, BGD, BTN, HND, NAM, IDN, TLS, MMR, FSM, IND	**Ccc**<br>GNQ, SDN, YEM, NGA, MRT
**Q4**			**D**<br>SWZ, GAB, NPL, KHM, STP, PAK, LAO, DJI, VUT, KIR	**Dd**<br>KEN, GHA, RWA, GMB, MLI, COG, TZA, AGO, COM, SLB, LBR, MWI, HTI, TGO, LSO, CMR, PNG, UGA, ZWE, SEN, SLE, BEN, BFA, MOZ, MDG, COD, ZMB, NER, ETH, ERI, BDI, CIV, SSD, GIN, AFG, TCD, GNB, SOM, CAF

Table S11. Classification of countries based on the quartiles (Q_1, Q_2, Q_3, Q_4) of the overall SDGGIS distribution and on the development community membership.

	I	II	III	IV
Q_1	**A**<br>DNK, SWE, FIN, FRA, AUT, DEU, CZE, NOR, NLD, EST, NZL, SVN, GBR, ISL, JPN, BEL, CHE, KOR, IRL, CAN, ESP, HRV, BLR, LVA, HUN, PRT, SVK, MLT, POL, ITA, LTU, LUX, USA, BGR, AUS, CHN	**Aa**<br>CHL, CRI, MDA, THA, UKR		
Q_2	**B**<br>ROU, SRB, ISR, GRC, RUS, CYP, SGP	**Bb**<br>URY, ARG, ECU, MDV, PER, VNM, CUB, BRA, IRN, AZE, ALB, TUN, DOM, ARE, COL, MYS, BIH, MKD, GEO, JAM, ARM, BHR, KAZ, MEX, TUR	**Bbb**<br>KGZ, UZB, DZA, FJI, TJK, MAR, BOL, JOR	
Q_3		**C**<br>OMN, TTO, MNE, SUR, SLV, PAN, QAT, LKA, LBN, SAU, MUS, KWT, VEN	**Cc**<br>NIC, BTN, PRY, EGY, STP, CPV, PHL, GAB, MNG, TKM, IDN, NPL, HND, BLZ, MMR, LAO, KHM, ZAF, GUY, IND, BGD, IRQ, VUT, NAM, BWA	**Ccc**<br>GHA, ZWE
Q_4			**D**<br>GTM, SYR, PAK, SWZ, DJI	**Dd**<br>SEN, KEN, RWA, CMR, TZA, CIV, GMB, COG, YEM, MRT, ETH, MOZ, COM, GIN, ZMB, UGA, BFA, PNG, TGO, BDI, MWI, SDN, AGO, LSO, BEN, MLI, AFG, NER, SLE, HTI, LBR, MDG, NGA, COD, TCD, CAF

References

1. ISO 3166 COUNTRY CODES – the International Standard for country codes and codes for their subdivisions. https://www.iso.org/iso-3166-country-codes.html (2013). Accessed: 2020-05-26.

2. World Development Indicators – The World Bank Group. http://datatopics.worldbank.org/world-development-indicators/ (2020). Accessed: 2020-05-26.

3. World Development Indicators – Data Catalog. https://datacatalog.worldbank.org/dataset/world-development-indicators/ (2020). Accessed: 2020-04-15.

4. Reichardt, J. & Bornholdt, S. Statistical Mechanics of Community Detection. *Phys. Rev. E* **74**, 016110 (2006).

5. Traag, V. A. & Bruggeman, J. Community detection in networks with positive and negative links. *Phys. Rev. E* **80**, 036115 (2009).

6. Traag, V. A., Waltman, L. & van Eck, N. J. From Louvain to Leiden: guaranteeing well-connected communities. *Sci. Rep.* **9**, 5233 (2019).

7. United Nations – Department of Economic and Social Affairs – World Population Prospects 2019, definition of regions. https://population.un.org/wpp/DefinitionOfRegions/ (2019). Accessed: 2020-05-26.

8. Yale Center for Environmental Law and Policy - YCELP - Yale University, Yale Data-Driven Environmental Solutions Group - Yale University, Center for International Earth Science Information Network - CIESIN - Columbia University, and World Economic Forum - WEF. 2018 Environmental Performance Index (EPI). Palisades, NY: NASA Socioeconomic Data and Applications Center (SEDAC). https://doi.org/10.7927/H4X928CF (2018). Accessed: 2020-05-26.

9. Wendling, Z. A. *et al.* 2018 Environmental Performance Index. New Haven, CT: Yale Center for Environmental Law & Policy. https://epi.yale.edu/ (2018). Accessed: 2020-02-20.

10. GBD 2016 Healthcare Access and Quality Collaborators. Measuring performance on the Healthcare Access and Quality Index for 195 countries and territories and selected subnational locations: a systematic analysis from the Global Burden of Disease Study 2016. *Lancet* **391**, 2236–2271 (2018).

11. Sachs, J., Schmidt-Traub, G., Kroll, C., Lafortune, G. & Fuller, G. *Sustainable Development Report 2019* (Bertelsmann Stiftung and Sustainable Development Solutions Network (SDSN), New York, 2019).
